# Discovery of Pancreatic Ductal Adenocarcinoma-Related Aberrant Glycosylations: A Multilateral Approach of Lectin Microarray-Based Tissue Glycomic Profiling With Public Transcriptomic Datasets

**DOI:** 10.3389/fonc.2020.00338

**Published:** 2020-03-13

**Authors:** Takanori Wagatsuma, Chiaki Nagai-Okatani, Atsushi Matsuda, Yohei Masugi, Masako Imaoka, Ken Yamazaki, Michiie Sakamoto, Atsushi Kuno

**Affiliations:** ^1^Project for Utilizing Glycans in the Development of Innovative Drug Discovery Technologies, Japan Bioindustry Association (JBA), Tokyo, Japan; ^2^Center for Integrated Medical Research, Keio University School of Medicine, Tokyo, Japan; ^3^Glycoscience and Glycotechnology Research Group, Biotechnology Research Institute for Drug Discovery, National Institute of Advanced Industrial Science and Technology, Ibaraki, Japan; ^4^Department of Biochemistry, Keio University School of Medicine, Tokyo, Japan; ^5^Department of Pathology, Keio University School of Medicine, Tokyo, Japan

**Keywords:** multi-omics, glycosylation, lectin microarray, ABO blood group antigen, The Cancer Genome Atlas (TCGA), Genotype-Tissue Expression (GTEx), glycosyltransferase, basigin/CD147

## Abstract

Aberrant protein glycosylation is one of the most notable features in cancerous tissues, and thereby glycoproteins with disease-relevant glycosylation alterations are fascinating targets for the development of biomarkers and therapeutic agents. For this purpose, a reliable strategy is needed for the analysis of glycosylation alterations occurring on specific glycoproteins during the progression of cancer. Here, we propose a bilateral approach combining lectin microarray-based tissue glycomic profiling and database-derived transcriptomic datasets. First, lectin microarray was used to perform differential glycomic profiling of crude extracts derived from non-tumor and tumor regions of frozen tissue sections from pancreatic ductal adenocarcinoma (PDAC). This analysis revealed two notable tissue glycome alterations in PDAC samples: increases in sialylated glycans and bisecting *N*-acetylglucosamine and a decrease in ABO blood group antigens. To examine aberrations in the glycosylation machinery related to these glycomic alterations, we next employed public datasets of gene expression profiles in cancerous and normal pancreases provided by The Cancer Genome Atlas and the Genotype-Tissue Expression projects, respectively. In this analysis, glycosyltransferases responsible for the glycosylation alterations showed aberrant gene expression in the cancerous tissues, consistent with the tissue glycomic profiles. The correlated alterations in glycosyltransferase expression and tissue glycomics were then evaluated by differential glycan profiling of a membrane *N*-glycoprotein, basigin, expressed in tumor and non-tumor pancreatic cells. The focused differential glycomic profiling for endogenous basigin derived from non-tumor and cancerous regions of PDAC tissue sections demonstrated that PDAC-relevant glycan alterations of basigin closely reflected the notable features in the disease-specific alterations in the tissue glycomes. In conclusion, the present multi-omics strategy using public transcriptomic datasets and experimental glycomic profiling using a tiny amount of clinical specimens successfully demonstrated that basigin is a representative *N*-glycoprotein that reflects PDAC-related aberrant glycosylations. This study indicates the usefulness of large public data sets such as the gene expression profiles of glycosylation-related genes for evaluation of the highly sensitive tissue glycomic profiling results. This strategy is expected to be useful for the discovery of novel glyco-biomarkers and glyco-therapeutic targets.

## Introduction

Protein glycosylation is one of the most important posttranslational modifications in eukaryotes. This modification is involved in fundamental biological processes by regulating the functions and localizations of glycan-attached proteins via modulations of their tertiary structure and interaction with other molecules (1). Glycosylation states, including the glycan structure attached to each glycosylation site of a protein, are altered by abnormal changes in the glycosylation machinery, including altered expression of glycosyltransferases. These alterations have been shown to be closely related to the development and progression of various diseases. The presence and significance of aberrant glycosylations have been recognized in cancer, as glycans have important roles in cell signaling and communication, tumor cell dissociation and invasion, cell-matrix interactions, tumor angiogenesis, immune modulation, and metastasis formation (2, 3). Accordingly, glycoproteins with disease-relevant glycosylation changes are fascinating targets for the development of novel biomarkers and therapeutic agents (4, 5). To efficiently accelerate this development, a reliable strategy is necessary for the analysis of glycosylation alterations occurring on a specific glycoprotein during disease progression (6, 7).

Lectin microarray was developed as a simple and reliable method for glycan analysis (8, 9). This technology allows researchers to obtain global glycomic profiles of *N*- and *O*-glycoproteins in biological samples, and these profiles are presented as the signal patterns of interactions between multiple lectins and carbohydrates (6, 10, 11). Taking advantage of the high sensitivity, this technology is fit to meet the aforementioned demands, and thus has been applied to spatial analysis of tissue sections to compare the glycomic profiles of different regions such as tumor and non-tumor regions (12–14). For the discovery of disease-specific glycosylation alterations, the feasibility and utility of this tissue glycomic profiling has been demonstrated using diseased tissues (15–21). Since lectin microarray can be used for glycan analysis not only of crude samples but also of target glycoproteins enriched by immunoprecipitation (22), this technology has been utilized for the verification of disease-relevant alterations of glycans attached to glyco-biomarker candidates (23–26).

Glycosylation-related genes including glycosyltransferases are known to exhibit aberrant expression patterns regulated by epigenetic mechanisms in association with diseases including cancer (27). Thus, disease-relevant alterations in gene expression profiles provide valuable information for the prediction of glycan structures. This prediction may be supported by constructing pathway maps of glycan biosynthesis using bioinformatic approaches such as the Kyoto Encyclopedia of Genes and Genomes (KEGG) PATHWAY^1^. Recent advances in next-generation sequencing technologies, which allow rapid, quantitative, and large-scale measurements of gene expression, have gathered attention in the glycomics field. Currently, many datasets of comprehensive gene expression profiles obtained by RNA sequencing technology are available as free-access databases. Among these databases, the Cancer Genome Atlas (TCGA) provides transcriptome profiles of more than 30 types of tumor and tumor-adjacent normal tissues (28), whereas the Genotype-Tissue Expression (GTEx) project provides transcriptomic data of healthy tissues from autopsies (29). The utility of integrative comparison of transcriptomic profiles obtained from the TCGA and GTEx databases has been demonstrated, revealing that normal tissue adjacent to the tumor represents a unique intermediate state between healthy and tumor (30).

Here, we evaluated the utility of a bilateral approach using a combination of lectin microarray-based tissue glycomic profiling and database-derived transcriptomic data. With this approach, we aimed to elucidate glycosylation alterations related to pancreatic ductal adenocarcinoma (PDAC), which is a highly chemoresistant cancer with poor prognosis (31). We first performed differential glycomic profiling of tumor and non-tumor regions within PDAC tissue sections to characterize PDAC-specific glycosylations. Next, we assessed the experimental results of the glycomic profiling by using the public databases to find the expression profiles of glycosyltransferases that may be responsible for the glycosylation alterations. Finally, we examined whether glycan changes observed in the glycome were reflected in the glycan profile of a specific glycoprotein using serial tissue sections. These experiments allowed us to evaluate the usefulness of moving back and forth between glycomic profiling analysis and transcriptomic analysis for efficient characterization of glycosylation alterations occurring on a specific glycoprotein during disease progression.

## Materials and Methods

### Clinical Specimens

Surgically resected frozen tissues from 14 PDAC patients were prepared and stored at −80^o^C. The study protocol conformed to the ethical guidelines of the 1975 Declaration of Helsinki and was approved by the ethics committee of Keio University School of Medicine (No. 20040034). Informed consent for the use of clinical specimens was obtained from all patients.

### Histochemical Analyses of Tissue Sections

Immunohistochemical staining for basigin of formalin-fixed paraffin-embedded (FFPE) tissue sections (5 μm thickness) was carried out using an automated staining system (Bond Max; Leica Biosystems, Wetzlar, Germany) as described previously (32). Tissue sections were deparaffinized, heated in ER2 solution (equivalent to Tris-EDTA buffer, pH 9.0; Leica Biosystems) at 100°C for 20 min to retrieve the epitope, and immunostained with an anti-basigin monoclonal antibody (10 μg/mL; Clone MEM-M6/1; Abcam, Cambridge, UK) using the Bond Polymer Refine Detection Kit (Leica Biosystems). Bright-field images of the stained sections were obtained using a digital pathological image scanner (NanoZoomer-XR C12000-03; Hamamatsu Photonics, Hamamatsu, Japan) with a 20× objective lens. The areas for tissue collection were measured using an image viewer (NDP.view2; Hamamatsu photonics).

### Differential Glycomic Profiling of Tissue Sections Using Lectin Microarray

Lectin microarray-based tissue glycomic profiling of PDAC patient specimens was performed as previously described (12), with the noted changes. Unstained PDAC frozen tissue sections (10 μm thickness) were compared to hematoxylin and eosin-stained serial sections and tissue fragments were collected from an area of 100–150 mm^2^. Membrane proteins were extracted using the CelLytic MEM Protein Extraction Kit (Sigma-Aldrich, St. Louis, MO, USA). The diluted aliquots (corresponding to 1 mm^2^) of the membrane protein fractions were labeled with 10 μg protein equivalent of Cy3 mono-reactive NHS ester (GE Healthcare, Buckinghamshire, UK) in a 10 μL volume for 1 h at room temperature in the dark. After the incubation, the fluorescently labeled solution was adjusted to 100 μL with a probing buffer (Tris buffered saline [TBS] containing 500 mM glycine, 1 mM CaCl_2_, 1 mM MnCl_2_, and 1% Triton X-100) and then incubated for 2 h at room temperature in the dark, for complete masking of the non-reacting Cy3-dye. After the masking, 10 μL (corresponding to 0.1 mm^2^) of the Cy3-labeled protein solution was diluted with phosphate buffered saline containing 1% Triton X-100 (PBSTx) up to 60 μL, and this sample was used for lectin microarray analysis. The adjusted Cy3-labeled protein samples were applied to a lectin microarray slide (LecChip^TM^ Ver.1.0; GlycoTechnica Ltd., Yokohama, Japan) prewashed with the probing buffer and incubated for overnight at 20°C with gently shaking to completely form the lectin–glycoprotein complex. After the incubation, the LecChip was washed with the PBSTx and then scanned by an evanescent-field fluorescence scanner (GlycoStation^TM^ Reader 1200, GlycoTechnica) in appropriate conditions. The fluorescence signal intensities were calculated using the image analyzer software (GlycoStation^TM^ ToolsPro Suite ver.1.5, GlyoTechnica). Finally, the mean lectin signals of triplicate spots were normalized by the mean value of 45 lectins immobilized on the array, and the mean-normalized signal intensities were used as the glycomic profiles for statistical analysis. The abbreviations and carbohydrate specificities of the 45 lectins are listed in Supplementary Table 1.

### Immunoprecipitation and Western Blotting Analysis of Basigin

Endogenous basigin was immunopurified from the membrane protein fraction extracted from the unstained PDAC frozen sections (10 μm thickness). Briefly, the anti-basigin antibody was biotinylated using the Biotin Labeling Kit-NH_2_ (Dojindo Laboratories, Kumamoto, Japan). Basigin was captured from the membrane protein fractions (corresponding to 20–40 mm^2^) using the biotinylated antibody (2.0 μg)-conjugated magnetic beads (Dynabeads MyOne Streptaidin T1, 100 μg; Life Technologies, Carlsbad, CA, USA) in TBS containing 1% Triton X-100 (TBSTx) overnight at 4°C with vigorous shaking. After the incubation, the beads were washed with TBSTx, resuspended in 10 μL of an elution buffer (TBS containing 0.2% sodium dodecyl sulfate), and incubated for 5 min at 95°C. Subsequently, the eluate was incubated with streptavidin-coated beads (100 μg) for 2 h at 4°C with vigorous shaking to remove the biotinylated antibody. Finally, the supernatant was collected as the heat-denatured eluate to use for further analyses.

The basigin was quantified by western blot analysis as follows. Proteins in the heat-denatured eluate (corresponding to 10 mm^2^) were separated by sodium dodecyl sulfate-polyacrylamide gel electrophoresis under non-reducing condition using SuperSep Ace (FUJIFILM Wako Pure Chemical, Osaka, Japan), and then transferred to a polyvinylidene difluoride membrane (Trans-Blot Turbo Mini PVDF Transfer Packs; BIO-RAD, Hercules, CA, USA). The membrane was blocked with a blocking reagent (Block Ace; DS Pharma Biomedical Co., Ltd., Osaka, Japan) for 1 h at 37°C, washed with TBS containing 0.1% Tween-20 (TBST), and then incubated with the biotinylated anti-basigin antibody (0.5 μg/mL) in TBST overnight at 4°C with gentle shaking. After washing with TBST, the membrane was incubated with streptavidin conjugated with horseradish peroxidase (1/100,000 diluted in TBST; Jackson ImmunoResearch Laboratories, Inc., West Grove, PA, USA) for 1 h at room temperature. After washing with TBST, basigin on the membrane was detected with a chemiluminescence substrate (ImmunoStar LD; FUJIFILM Wako Pure Chemical) using a chemiluminescent scanner (C-DiGit; LI-COR, Inc., Lincoln, NE, USA).

### Glycan Profiling of Basigin by Antibody-Overlay Lectin Microarray

To obtain the glycan profile of basigin, antibody-overlay lectin microarray was performed as described previously (22) with the changes described here. Briefly, 10 μL of the heat-denatured eluate (corresponding to 10 mm^2^) prepared as described above was diluted and applied to the LecChip and incubated overnight at 20°C in a similar manner as the tissue glycomic profiling. After the incubation, 20 μg of human serum IgG (Sigma-Aldrich) was added to each well to block the interaction of free lectins with detection antibody for 30 min at 20°C. After washing with PBSTx, the LecChip was incubated with the biotinylated anti-basigin antibody (100 ng) for 1 h at 20°C. After washing with PBSTx, the LecChip was further incubated with Cy3-conjugated streptavidin (200 ng; GE Healthcare) for 20 min at 20°C. After washing with PBSTx, the LecChip was scanned using GlycoStation Reader 1200. The data processing was performed as described above to obtain the mean-normalized signal intensities as the glycan profiles of basigin (25). It should be noted that the normalized values of the glycan profiling data for basigin could not be compared between those of the tissue glycomic profiling data, owing to the batch-to-batch variations of the lectin array chips used for these analyses.

### Gene Expression Analysis Using the Public Databases

To compare the gene expression levels using the data from the TCGA and GTEx databases, we used the University of California Santa Cruz (UCSC) Xena Browser^2^ as described previously (33). Briefly, the expected count values of RNA sequencing by Expectation-Maximization (RSEM) for genes of interest were normalized using DESeq2 (version 2018-05-08) to remove the batch effect, and these calculated values were downloaded from the Xena Browser. The datasets of RSEM expected counts of cancerous pancreases (*N* = 178) and normal pancreases (*N* = 165) were obtained from the TCGA and GTEx databases, respectively.

### Statistics

Wilcoxon matched-pairs signed rank test was used for comparing the glycomic profiles between tumor and non-tumor regions and comparing the gene expression levels between cancerous and normal pancreases. Fold change between two samples was calculated using the median values and presented as the absolute log2 value of tumor/non-tumor or cancerous/normal ratio. The calculation was performed using GraphPad Prism 8 for macOS (GraphPad Software, San Diego, CA). The differences with *P* < 0.05 and *P* = 0.0001 were considered significant and extremely significant, respectively. As multivariate analysis, principle component analysis (PCA) was performed using JMP 14.2.0 (SAS Institute Inc., Cary, NC).

## Results

### Lectin Microarray Analysis of Tumor and Non-tumor Regions of PDAC Tissue Sections

**Figure 1:**
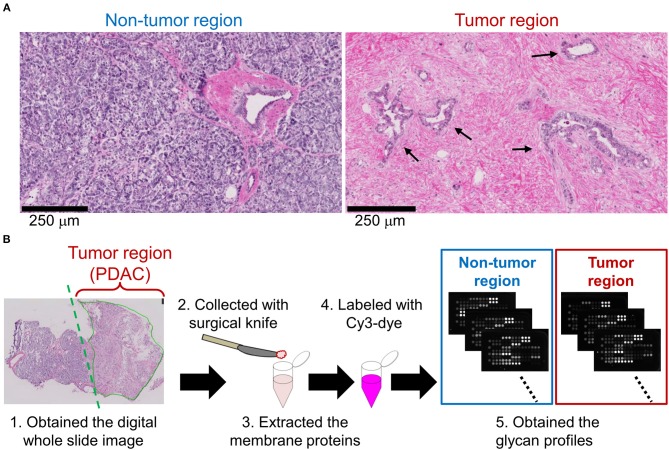
Differential glycomic profiling of PDAC tissue sections. **(A)** Representative images of non-tumor and tumor regions in hematoxylin and eosin-stained frozen sections. The arrows indicate the cancerous ducts. **(B)** Schematic overview of lectin microarray-based glycomic profiling of unstained frozen sections.

We first aimed to elucidate PDAC-related alterations in protein glycosylations. Based on the observation of hematoxylin and eosin-stained frozen sections of PDAC patients, tumor regions were distinguishable from non-tumor regions by the typical pathological characteristics. In the non-tumor regions, pancreatic parenchymal cells called acinar cells existed in a highly dense and organized state (Figure 1A, left). In contrast, in tumor regions, irregular ductal structures with diffuse distribution were observed and the acinar cells were replaced by remarkable fibrosis (Figure 1A, right). Using 14 cases of PDAC frozen sections with the pathomorphological features shown in Supplementary Table 2, lectin microarray was performed (Figure 1B). Briefly, tissue fragments were collected from the non-tumor and PDAC regions of each frozen section. The membrane proteins were extracted and subjected to lectin microarray analysis to obtain tissue glycomic profiles of the clinical specimens. In the tissue glycomic profiles of the 14 PDAC cases (Supplementary Figure 1), all *O*-glycan binders (e.g., Jacalin and ACA) showed relatively low signals, indicating a low abundance of these glycan structures in the tissue glycomes of both the tumor and non-tumor regions. Notably, the signals of several lectins for ABO blood group antigens (EEL, DBA, PTL-I, UEA-I, and TJA-II) were detected only in the non-tumor regions, suggesting this is a PDAC-relevant alteration in the tissue glycome.

### Differential Tissue Glycomic Profiling Between the Two Regions by Univariate Analysis

**Figure 2:**
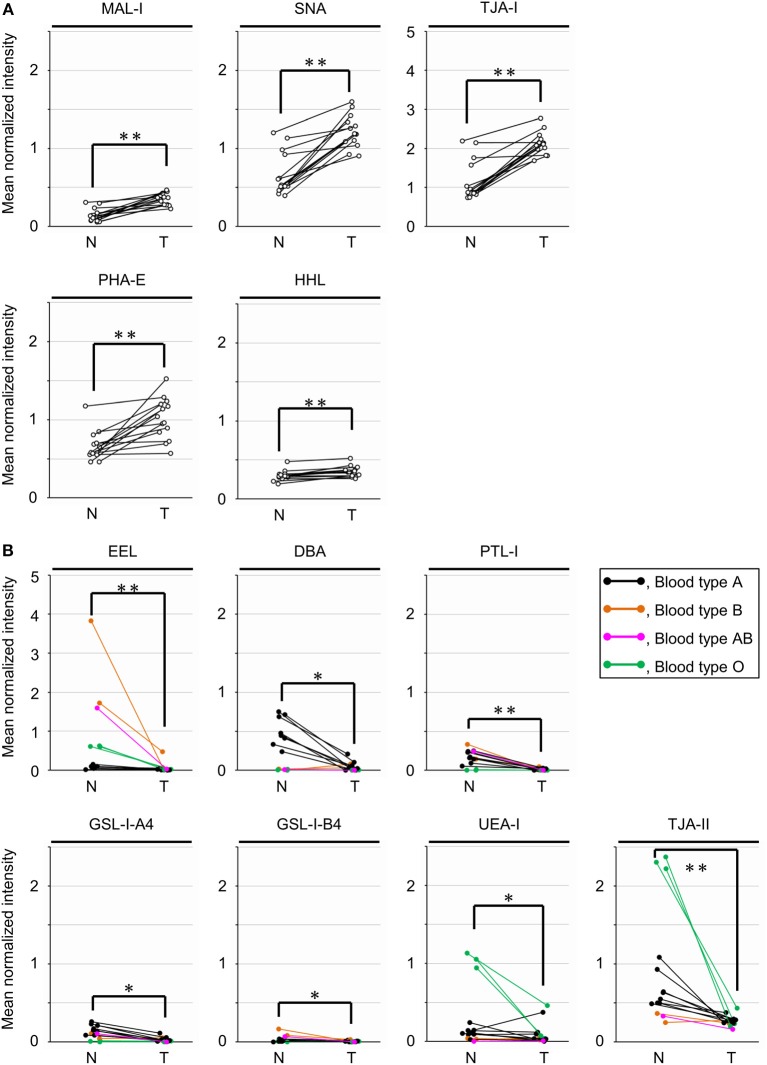
Univariate analysis of the tissue glycomic profiles obtained from the non-tumor (N) and tumor (T) regions of PDAC tissue sections. **(A)** Comparison of the relative signal intensities of the five lectins that showed extremely significant higher signals (*P* = 0.0001). **(B)** Comparison of the relative signal intensities of the seven lectins recognizing ABO blood group antigens. *N* = 14 for each region. *P < 0.05, **P = 0.0001. The median and *P*-values are summarized in Table 1.

To objectively compare the lectin signals between the PDAC regions and the case-matched non-tumor regions, we analyzed the lectin microarray data by univariate analysis for all 45 lectins on the array (Supplementary Table 1). The PDAC samples showed higher signals (*P* = 0.0001) for five lectins recognizing α2,3-sialic acid (MAL-I), α2,6-sialic acid (SNA and TJA-I), bisecting *N*-acetylglucosamine (GlcNAc; PHA-E), and high mannose (HHL) (Figure 2A and Table 1). In contrast, significant lower signals (*P* = 0.0001) were observed for one lectin recognizing the B blood group antigen (EEL) (Figure 2B and Table 1). Since the difference in the EEL signals between the tumor and non-tumor regions was large, with the absolute log2 fold change (|FC[log2]|; tumor/non-tumor ratio) of 2.9, we further analyzed the other lectins recognizing ABO blood group antigens. The signal intensities of DBA and GSL-I-A4 (for the A blood group antigen), PTL-I (for the A and B blood group antigens), GSL-I-B4 (for the B blood group antigen), and UEA-I and TJA-II (for the O blood group antigen [H antigen]) were different between these two regions with |FC(log2)| > 1 and *P* < 0.05 (Figure 2B and Table 1). Notably, the signal intensities of these seven lectins for ABO blood group antigens were also different in the non-tumor samples according to the blood types of the patients.

**Table 1:** Summary of univariate analysis for tissue glycomic profiles of tumor (T) and non-tumor (N) regions.

**Lectin**	***P***	**Median (N)**	**Median (T)**	**|FC(log2[T/N])|^a^**	**Summary**
MAL-I	0.0001	0.13	0.36	**1.5**	Up
SNA	0.0001	0.54	1.19	**1.1**	Up
TJA-I	0.0001	0.88	2.12	**1.3**	Up
PHA-E	0.0001	0.58	1.00	0.8	Up
HHL	0.0001	0.29	0.35	0.2	Up
MPA	0.0002	0.09	0.14	0.6	Up
SSA	0.0004	0.62	1.30	**1.1**	Up
ACG	0.0006	2.31	2.73	0.2	Up
TxLC-I	0.0067	0.15	0.21	0.5	Up
EEL	0.0001	0.12	0.02	**2.9**	Down
TJA-II	0.0002	0.59	0.26	**1.2**	Down
WFA	0.0004	0.24	0.07	**1.7**	Down
PTL-I	0.0005	0.15	0.00	**5.1**	Down
Calsepa	0.0012	0.80	0.61	0.4	Down
GSL-I-A4	0.0017	0.11	0.01	**2.9**	Down
DSA	0.0031	4.22	3.83	0.1	Down
SBA	0.0031	0.06	0.00	**3.7**	Down
GSL-I-B4	0.0052	0.01	0.00	**1.5**	Down
DBA	0.011	0.29	0.02	**4.1**	Down
UEA-I	0.030	0.11	0.04	**1.5**	Down
LCA	0.042	0.54	0.45	0.3	Down
UDA	0.049	6.59	5.63	0.2	Down
NPA	0.058	1.60	1.18	0.4	–
LEL	0.058	5.89	6.34	0.1	–
Jacalin	0.058	0.42	0.47	0.1	–
STL	0.091	3.72	3.82	0.0	–
PWM	0.091	0.04	0.02	0.7	–
BPL	0.10	0.09	0.13	0.5	–
PSA	0.12	0.31	0.34	0.1	–
ECA	0.12	0.24	0.13	0.9	–
GNA	0.12	0.50	0.42	0.3	–
ABA	0.14	1.01	0.81	0.3	–
MAH	0.24	0.23	0.24	0.0	–
GSL-II	0.33	0.04	0.02	0.8	–
LTL	0.43	0.05	0.06	0.2	–
WGA	0.43	1.90	1.80	0.1	–
VVA	0.50	0.00	0.00	N.D.	–
ACA	0.54	0.31	0.34	0.1	–
AOL	0.58	1.00	1.00	0.0	–
ConA	0.58	2.57	2.25	0.2	–
AAL	0.76	1.62	1.45	0.2	–
PNA	0.81	0.00	0.00	N.D.	–
PHA-L	0.95	0.12	0.12	0.0	–
RCA120	> 0.99	1.74	1.74	0.0	–
HPA	> 0.99	0.00	0.00	N.D.	–

a *The significant changes (|FC(log2[T/N])| > 1) are highlighted in bold. N.D., not determined due to the zero values for the non-tumor samples*.

### Differential Tissue Glycomic Profiling by Multivariate Analysis

**Figure 3:**
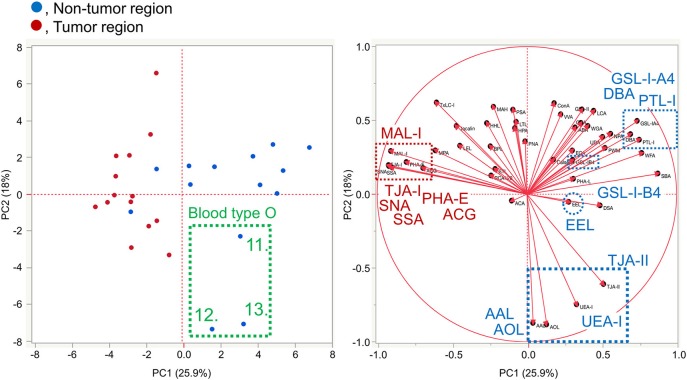
Multivariate analysis of the tissue glycomic profiles by PCA. In the score plot (**left panel**), the lectin microarray data of the non-tumor (blue) and tumor (red) regions of PDAC tissue sections (*N* = 14) were plotted. The numbers of three non-tumor samples obtained from O blood group patients (No. 11–13) are indicated. In the loading plot (**right panel**), the relative signal intensities of 45 lectins on the array are plotted. The lectins extracted for the non-tumor and tumor regions are indicated in blue and red, respectively.

To further investigate the differences in the tissue glycomic profiles of PDAC and case-matched non-tumor regions, PCA was performed using the lectin microarray data. This multivariate analysis could distinguish between the tumor and non-tumor regions based on the PC1 axis (Figure 3). Sialic acid-binding lectins (i.e., lectins recognizing α2,3-sialic acid [MAL-I and ACG]) and α2,6-sialic acid [SNA, SSA, and TJA-I]) and a bisecting GlcNAc-binding lectin (PHA-E) were extracted for characterizing the tumor regions. The lectins for A and B blood group antigens (PTL-I, DBA, and EEL) were extracted for the non-tumor regions. Based on the PC2 axis, normal specimens were further separated into two groups, one of which was categorized by the blood type O group (Patient No. 11–13). In these three patients, relatively higher signals were observed for a lectin for H antigen (TJA-II) and lectins that bind to fucose residues including α1,2-fucose configuring H antigen (UEA-I, AAL, and AOL). Combined with the univariate analysis results, the increase in sialylated glycans and bisecting GlcNAc and the decrease in the ABO blood group antigens were notable features in PDAC-relevant alterations in the tissue glycome.

### Comparison of the Gene Expression Levels of Selected Glycosyltransferases Using Public Datasets

**Figure 4:**
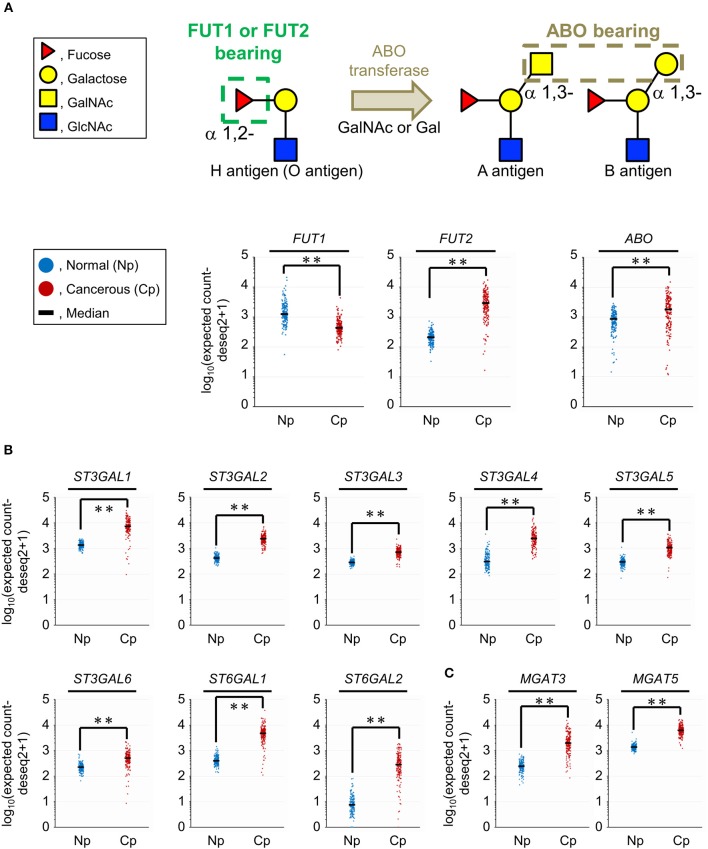
Comparison of the gene expression levels of selected glycosyltransferases between the normal (Np) and cancerous (Cp) pancreases. **(A)** Glycan structures of the O, A, and B blood group antigens (upper panel) and gene expression levels of their related glycosyltransferases (lower panels). **(B)** Gene expression levels of sialyltransferases related to α2,3-, and α2,6-sialylation of terminal galactose residues. **(C)** Gene expression levels of mannosyl-glycoprotein *N*-acetylglucosaminyltransferases related to the biosynthesis of bisecting GlcNAc. *N* = 165 for Np and *N* = 178 for Cp. **P = 0.0001.

The results of the tissue glycomic profiling suggested a possibility that the glycosylation machinery for the biosynthesis of ABO blood group antigens, sialylated glycans, and bisecting GlcNAc would be altered in the tissue glycome. To evaluate this hypothesis, we next used two public databases, GTEx and TCGA, to analyze the gene expression levels of glycosyltransferases responsible for these glycosylations. For the synthesis of the core structure (H antigen), FUT1 and FUT2 catalyze α1,2-fucosylation. The structural diversity of ABO blood group antigens is determined by the *ABO* gene. This gene encodes blood group-specific glycosyltransferase for addition of the determinant glycan (i.e., α1,3-*N*-acetylgalactosamine [GalNAc] for the blood group A and α1,3-galactose for the blood group B) to H antigen (Figure 4A). Therefore, we compared gene expression levels of these three glycosyltransferases between normal and cancerous pancreas tissues. The *FUT1* expression was decreased (*P* = 0.0001) in the cancerous tissues compared to the normal tissues with |FC(log2)| of 1.5, whereas the expression of *FUT2* and *ABO* was significantly increased (*P* = 0.0001) with |FC(log2)| of 3.8 and 1.1, respectively. Next, the gene expression of the sialyltransferases ST3GALs and ST6GALs, which are responsible for α2,3- and α2,6-sialylation of terminal galactose residues, respectively, was analyzed (Figure 4B). The gene expression levels of six ST3GALs (ST3GAL1–6) and two ST6GALs (ST6GAL1 and ST6GAL2) were significantly increased in the cancerous pancreases compared to the normal pancreases with |FC(log2)| > 1 and *P* = 0.0001. Regarding bisecting GlcNAc, gene expression of the synthesizing enzyme MGAT3 and its competitively acting enzyme MGAT5 were both increased in PDAC tissues (Figure 4C). The increased expression level of *MGAT3* was higher than that of *MGAT5* (|FC(log2)| of 3.0 for MGAT3 and 2.2 for MGAT5). Together, these results show that aberrant gene expression of glycosyltransferases observed in pancreatic cancer is consistent with the tissue glycomic profiles, and thus strongly supports the validity of the tissue glycomic profiling results.

### Expression and Localization of the Membrane *N*-Glycoprotein Basigin in PDAC Tissues

**Figure 5:**
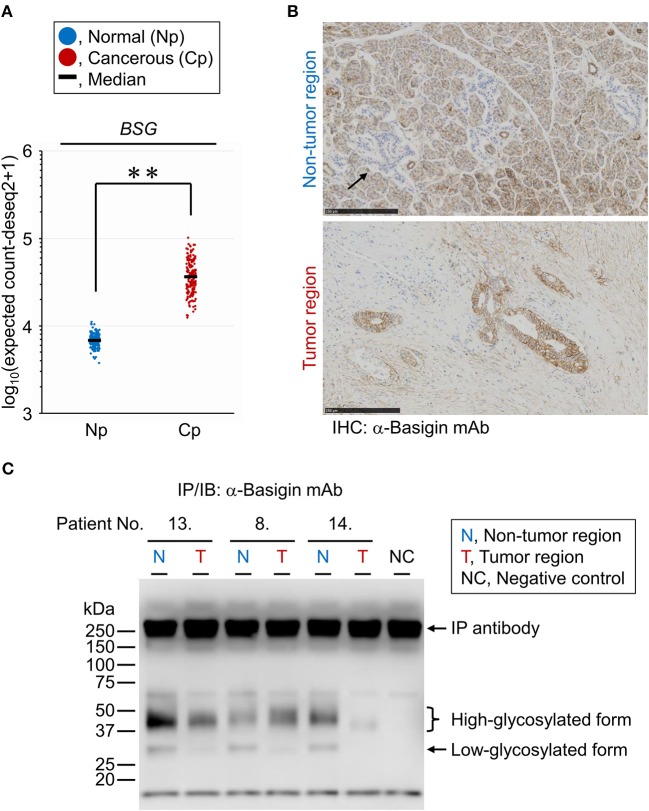
Expression analyses of endogenous basigin in PDAC tissues. **(A)** Expression levels of basigin-encoding gene (*BSG*) in normal (Np; *N* = 165) and cancerous (Cp; *N* = 178) pancreases. **P = 0.0001. **(B)** Representative images of the non-tumor (upper panel) and tumor (lower panel) regions of FFPE sections stained for basigin. The arrow indicates the islet of Langerhans, where basigin was not expressed. IHC, immunohistochemistry. **(C)** Western blot analysis for basigin immunoprecipitated from frozen tissues of representative PDAC cases. This *N*-glycoprotein was detected as high- and low-glycosylated forms. IP, immunoprecipitation; IB, immunoblot.

The PDAC-related changes seen in the tissue glycome are likely a consequence of coordinated alterations occurring in individual membrane glycoproteins due to the aberrations in the glycosylation machinery of the host cells. To verify whether the glycan profile of a specific glycoprotein reflects the glycomic features of the tissue crude glycoproteins, we performed differential glycan profiling of a membrane *N*-glycoprotein, basigin (also referred to as CD147). We selected basigin as a model glycoprotein because this type I single-pass transmembrane glycoprotein is expressed in a variety of organs and tissues including the pancreas, and *N*-glycosylation of the three sites has been demonstrated (34). Using peptide:*N*-glycanase, we confirmed that the glycosylated forms of basigin expressed in the human PDAC cell line CFPAC-1 were only decorated by *N*-glycans (Supplementary Figure 2). We confirmed the changes in the expression level of basigin-encoding gene (*BSG*) in PDAC using public databases, as described above for the glycosyltransferases. The *BSG* expression levels were increased significantly in the cancerous pancreases compared to the normal tissues with |FC(log2)| of 2.4 and *P* = 0.0001, although this gene was abundantly expressed even in the normal tissues (Figure 5A). The localization of basigin was also examined in PDAC specimens by immunohistochemistry with an anti-basigin antibody. In the non-tumor regions, basigin expressed in ductal cells and acinar cells but not in Langerhans cells (Figure 5B, upper panel). In the tumor regions, much higher expression of basigin was observed on the cell membranes of the cells on the circumference of the cancerous ducts (Figure 5B, lower panel).

### Lectin Microarray Analysis of Endogenous Basigin Enriched From PDAC Tissue Sections

For differential glycan profiling of endogenous basigin derived from the tumor and non-tumor regions of PDAC tissues, we immunoprecipitated basigin from the matched-pair frozen sections of 14 PDAC cases that were serial sections of those used for the tissue glycomic profiling. The immunoprecipitation of the protein was confirmed by western blot analysis, in which basigin was detected in two primary forms: high-glycosylated (approximately 42 kDa) and low-glycosylated (approximately 30 kDa) (Figure 5C). Interestingly, basigin derived from the tumor regions was exclusively expressed as the high-glycosylated form due to the significant down-regulation of the low-glycosylated form. This analysis also confirmed the specificity of the anti-basigin antibody, as a negative control yielded the bands derived from the antibody, and all the six samples prepared from tumor and non-tumor tissues exhibited additional bands corresponding to basigin. Subsequent antibody-overlay lectin microarray analysis, which employed the same antibody to ensure the basigin-specific detection, successfully provided the signals, most of which were derived from basigin (Supplementary Figure 3). By this analysis, each glycan profile of basigin enriched from the tumor and non-tumor regions of the 14 cases was obtained (Supplementary Figure 4). No or low signals of lectins recognizing *O*-glycans (e.g., Jacalin and ACA) and high mannose (NPA, ConA, GNA, and HHL) were detected for basigin in both regions, suggesting that this glycoprotein was not decorated with these glycans. Notably, the seven lectins recognizing ABO blood group antigens (EEL, DBA, PTL-I, GSL-I-A4, GSL-I-B4, UEA-I, and TJA-II) were detected almost exclusively in the non-tumor regions, as is the case in the tissue glycomic profiles (Supplementary Figure 1). These results indicate that the PDAC-relevant alterations in the glycosylation machinery affected the glycan profile of endogenous basigin.

### Differential Glycan Profiling of Basigin Between the Two Regions by Univariate Analysis

**Figure 6:**
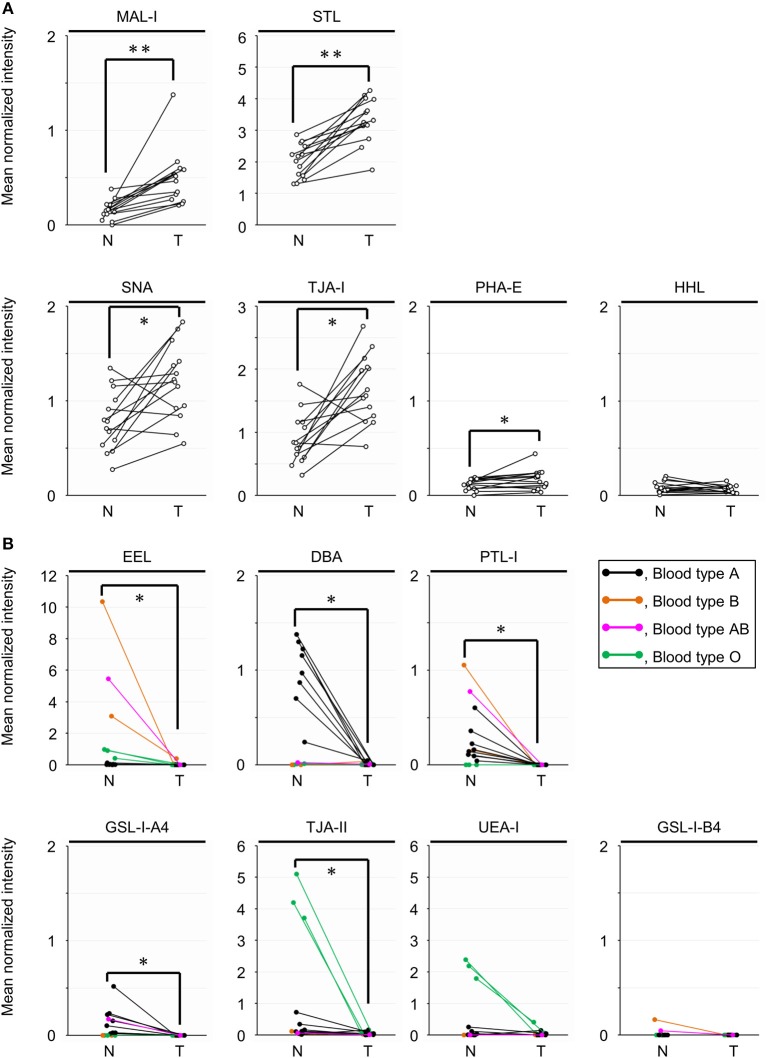
Univariate analysis of the glycan profiles of basigin derived from the non-tumor (N) and tumor (T) regions of PDAC tissue sections. **(A)** Comparison of the relative signal intensities of the six lectins that showed extremely significant differences (*P* = 0.0001) in the differential glycan profiling of basigin and/or in the differential tissue glycomic profiling (Figure 2A). **(B)** Comparison of the relative signal intensities of the seven lectins recognizing ABO blood group antigens. *N* = 14 for each region. *P < 0.05, **P = 0.0001. The median and *P*-values are summarized in Table 2.

To characterize PDAC-relevant glycan alterations of basigin, we analyzed the lectin microarray data by univariate analysis for the 45 lectins between the tumor and non-tumor regions, as with the differential tissue glycomic profiling. The extremely significant higher signals (*P* = 0.0001) were observed in the tumor regions for one lectin recognizing α2,3-sialic acid (MAL-I) and another lectin for (GlcNAc)_n_ and/or poly-*N*-acetyllactosamine (polyLacNAc; STL), where |FC(log2)| were 1.7 and 0.6, respectively (Figure 6A and Table 2). To compare features in the glycan profiles of basigin with those in tissue glycomic profiles, we focused on the lectins that showed differences in the differential tissue glycomic profiling. Among the five lectins that showed extremely significant higher signals (*P* = 0.0001; MAL-I, SNA, TJA-I, PHA-E, and HHL) in the glycomic profiling, four (HHL excluded) exhibited differences with *P* < 0.05 in basigin (Figure 6A and Table 2). Regarding the lectins recognizing ABO blood group antigens, the significant lower signals were detected in the tumor regions with *P* < 0.01 for EEL (for the B blood group antigen), DBA (for the A blood group antigen), PTL-I (for the A and B blood group antigens), GSL-I-A4 (for the A blood group antigen), and TJA-II (for H antigen), but not for UEA-I (for H antigen); the signal of GSL-I-B4 was not detected in either sample (Figure 6B and Table 2). The differences in the signals of these five lectins with *P* < 0.01 were notable, as |FC(log2)| was 6.1 for EEL, 9.2 for DBA, > 10 for PTL-I and GSL-I-A4, and 1.4 for TJA-II.

**Table 2:** Summary of univariate analysis for glycomic profiles of basigin from tumor (T) and non-tumor (N) regions.

**Lectin**	***P*^a^**	**Median (N)**	**Median (T)**	**|FC(log2[T/N])|^b^**	**Summary**
MAL-I	0.0001	0.15	0.49	**1.7**	Up
STL	0.0001	2.10	3.29	0.6	Up
LEL	0.0009	6.20	8.45	0.4	Up
TJA-I	0.0012	0.79	1.63	**1.0**	Up
ACG	0.0012	3.73	6.39	0.8	Up
SNA	0.0067	0.75	1.21	0.7	Up
BPL	0.017	0.01	0.03	**1.2**	Up
SSA	0.025	0.75	1.15	0.6	Up
RCA120	0.025	0.48	0.62	0.4	Up
PHA-E	0.025	0.12	0.17	0.5	Up
EEL	0.0002	0.08	0.00	**6.1**	Down
ConA	0.0004	0.30	0.18	0.7	Down
PTL-I	0.0010	0.15	0.00	**>10**	Down
DSA	0.0023	8.99	6.19	0.5	Down
GSL-I-A4	0.0039	0.03	0.00	**>10**	Down
TJA-II	0.0052	0.11	0.04	**1.4**	Down
DBA	0.0061	0.47	0.00	**9.2**	Down
WGA	0.0085	3.61	0.65	**2.5**	Down
Jacalin	0.098	0.00	0.00	N.D.	–
PHA-L	0.11	0.00	0.00	N.D.	–
MAH	0.11	0.00	0.00	N.D.	–
UEA-I	0.13	0.01	0.01	0.2	–
GNA	0.21	0.01	0.00	**3.3**	–
HHL	0.46	0.07	0.07	0.0	–
ACA	0.46	0.02	0.01	**1.6**	–
GSL-I-B4	0.50	0.00	0.00	N.D.	–
NPA	0.51	0.08	0.05	0.6	–
LTL	0.63	0.00	0.00	N.D.	–
GSL-II	0.65	0.00	0.00	N.D.	–
AAL	0.76	4.33	4.92	0.2	–
Calsepa	0.76	0.20	0.18	0.2	–
ECA	0.81	0.08	0.06	0.4	–
AOL	0.86	1.90	3.18	0.7	–
WFA	> 0.99	0.00	0.00	N.D.	–
SBA	> 0.99	0.00	0.00	N.D.	–
PWM	1.00	0.00	0.00	N.D.	–
PNA	1.00	0.00	0.00	N.D.	–
MPA	1.00	0.00	0.00	N.D.	–
HPA	1.00	0.00	0.00	N.D.	–
PSA	N.D.	0.45	0.63	0.5	–
LCA	N.D.	0.84	1.13	0.4	–
TxLC-I	N.D.	0.25	0.27	0.1	–
ABA	N.D.	0.86	0.83	0.1	–
UDA	N.D.	1.45	1.91	0.4	–
VVA	N.D.	0.11	0.19	0.8	–

a *N.D., not determined due to the high background caused by the detection antibody*.

b *The significant changes (|FC(log2[T/N])| > 1) are highlighted in bold. N.D., not determined due to the zero values for the non-tumor samples*.

### Differential Glycan Profiling of Basigin by Multivariate Analysis

**Figure 7:**
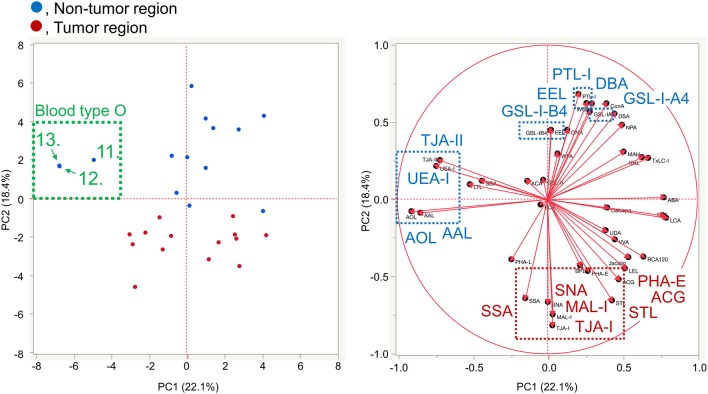
Multivariate analysis of the glycan profiles of basigin by PCA. In the score plot (**left panel**), the lectin microarray data of endogenous basigin enriched from the non-tumor (blue) and tumor (red) regions of PDAC tissue sections (*N* = 14) are plotted. The numbers of three non-tumor samples obtained from O blood group patients (No. 11–13) are indicated. In the loading plot (**right panel**), the relative signal intensities of 45 lectins on the array are plotted. The lectins extracted from the non-tumor and tumor regions are indicated in blue and red, respectively.

The lectin microarray data for basigin were also analyzed by PCA, as was done for the differential tissue glycomic profiling (Figure 3). The score plot shows that the glycomes of basigin are quite different between the non-tumor and PDAC regions based on the PC2 axis (Figure 7). The lectins recognizing α2,3-sialic acid (MAL-I and ACG), α2,6-sialic acid (SNA, SSA, and TJA-I), bisecting GlcNAc (PHA-E), and (GlcNAc)_n_ and polyLacNAc (STL) were extracted for characterizing the tumor samples. The lectins recognizing ABO blood group antigens such as DBA, PTL-I, and EEL were extracted for the non-tumor samples. Interestingly, as is the case in the tissue glycomic analysis, three non-tumor specimens of the blood type O group (Patient No. 11–13) were categorized into a different cluster from the other non-tumor specimens based on the PC1 axis. In these patients, the signals of the H antigen-binding lectin (TJA-II) and fucose-binding lectins (UEA-I, AAL, and AOL) were relatively higher, which is also similar to the results of tissue glycomic profiling. Combined with the univariate analysis results, these results demonstrate that the PDAC-relevant glycan alterations of a specific glycoprotein, basigin, reflect the notable features in the disease-specific alterations in the tissue glycomics, including the increases in sialic acid, bisecting GlcNAc, and the decrease in ABO blood group antigens.

## Discussion

The present study demonstrated that additional glycan profiling of a specific endogenous glycoprotein on pancreatic cancer cells could confirm the PDAC-relevant features identified by tissue glycomic and transcriptomic profiling. The results highlight the usefulness of this unique bilateral strategy with a “back and forth analysis” approach. In brief, the differential glycomic profiling of tissue membrane glycoproteins initially identified three notable features in the glycosylation alterations occurring in PDAC tissues: the increase in sialylated glycans, the increase in bisecting GlcNAc, and the decrease in ABO blood group antigens. These alterations in the tissue glycome were supported *in silico* by the subsequent gene expression analyses using public datasets of normal and PDAC tissues, in which the expression of glycosyltransferases responsible for the synthesis of these glycosylations were consistently altered. Finally, the differential glycan profiling of the membrane *N*-glycoprotein basigin, expressing in both tumor and non-tumor regions, definitively confirmed that the alterations were observed in a membrane protein on cancerous cells.

A distinctive feature of the present approach is the use of public datasets of tissue gene expression profiles for the *in silico* verification of lectin microarray-based tissue glycomic profiling. As described in the Introduction, currently emerging bioinformatic approaches using comprehensive transcriptomic datasets are considered useful for the prediction of the resulting glycan structures. However, disease-relevant aberrations in the glycosylation machinery involves not only the gene expression changes of glycosylation-related proteins but also other changes, such as their subcellular localization and the availability of sugar nucleotides (35). Owing to this complexity of the glycosylation machinery, it is indispensable to compare the tissue expression profiles of glycosylation-related genes with the glycan structures expressed in the diseased tissues as the end products of disease-relevant machinery changes, i.e., glycomics. Here, as an approach to tissue glycomic analysis, we employed a lectin microarray-based glycan profiling method for the comparison of non-tumor and PDAC regions in the same tissue section, which successfully provided information on PDAC-relevant glycan structures. Although this technology provides less detailed information on the glycan structures compared to mass spectrometry-based approaches (6), this technology has an advantage in that it allows highly sensitive differential glycan analysis in a simple and high-throughput way with statistical power. This advantage enables glycan profiling of a tiny amount of an endogenous glycoprotein specifically localized in the limited cells within the clinical tissue specimens, as demonstrated here with the membrane *N*-glycoprotein basigin. Regarding transcriptional analysis, in addition to the limitation of sample amount, we recognized a limitation of the quality of tissue samples for RNA sequencing in our preliminary experiments, probably due to an influence of endogenous RNases abundant in pancreatic tissues. These limitations of mass spectrometry-based and transcriptional analyses for clinical specimens highlight the usefulness of the present strategy, in particular for clinical and translational researches. Regarding a technical impact, this is the first report for the lectin microarray analysis of an endogenous *N*-glycoprotein. Previously this analysis could only be done using the method described for the glycan analyses of the *O*-glycoproteins in FFPE tissue sections (22, 25).

A high level of sialylated glycans, which is one of distinctive features of cancer cells (36), was successfully detected in the present tissue glycomic profiling, indicating the validity of these results. As abnormal sialylation is known to often be associated with malignant properties, including invasiveness and poor prognosis in patients (35, 37), this glycosylation aberration is a potential therapeutic target for pancreatic cancer (38). The present study showed increased levels of both α2,3- and α2,6-sialylation in the PDAC tissue membrane glycoproteome (Figure 2A), which is consistent with a previous study using human PDAC cell lines (39). Consistent with these PDAC-relevant glycomic features, the present gene expression analyses showed increased levels of both ST3GALs and ST6GALs (Figure 4B), suggesting coordinating regulation. Among these sialyltransferases, ST6GAL1 has a crucial role in angiogenesis in cancerous tissues (40), which protects tumor cells against hypoxia by enhancing HIF-1 signaling (41). Another study has reported a regulatory role for ST6GAL1 in the fructose-responsive invasiveness of PDAC (42). Consistent with these previous studies, we found that the increased level of ST6GAL1 in cancerous tissue was relatively large (Figure 4B). Notably, its counterpart α2,6-sialyltransferase for Galβ1,4GlcNAc-bearing carbohydrates, ST6GAL2, showed a greater increase in expression in the cancerous pancreases compared with ST6GAL1. Since these two ST6GALs have discrete carbohydrate preferences (43), the present results may suggest the importance of investigating potential functions of ST6GAL2 in tumor pathophysiology.

The present tissue glycomic profiling also revealed an increase in bisecting GlcNAc in the tumor regions of PDAC tissues, which has previously been reported in hepatoma, leukemia, and ovarian cancer (44), but not in pancreatic cancer. MGAT3 is the key enzyme for the biosynthesis of this branching sugar residue in *N*-glycans, an activity that is suppressed by MGAT5 function (44). Based on this relationship, the present results, showing a higher cancer/normal ratio of *MGAT3* expression compared with that of *MGAT5* expression (Figure 4C), are supportive evidence for the increase in bisecting GlcNAc in PDAC tissues. The alterations in gene expression and glycome levels are also consistent with a previous report showing the increased degree of MGAT3 and MGAT5 activities in the tissues of pancreatic carcinoma patients (45). Notably, a more recent study has demonstrated that the introduction of bisecting GlcNAc suppresses terminal modifications of *N*-glycans such as fucosylation and sialylation in genetically modified mice (46). The present tissue glycomic profiling results are consistent with this previous report in that the increased level of bisecting GlcNAc was accompanied by decreased fucosylation. In contrast, the present results appear inconsistent with the previous report in that both bisecting GlcNAc and sialic acid were increased in the PDAC tissues. This apparent contradiction may suggest that these glycosylation alterations occur in discrete cells within the tumor tissues.

Here, decreased levels of ABO blood group antigens in the tissue glycoproteome were identified as one of the notable PDAC-relevant alterations. Importantly, similar glycosylation alteration was observed in the glycan analysis of the membrane *N*-glycoprotein basigin. This observation could confirm that the tissue glycomic profiling result is surely attributed to changes in glycan structures attached to tissue-derived glycoproteins, excluding a potential influence of contaminated blood components. The decreased levels of ABO blood groups antigens were shown as the significantly decreased signals of the seven lectins recognizing these antigens in PDAC samples, Among these lectins, UEA-I exclusively prefers to H type 2 antigens but do not binds to H type 1 antigens, whereas EEL, DBA, and PTL-I bind to both H type 1 and H type 2 antigen and their derivatives (e.g., A and B antigens). Considering this carbohydrate specificity, the present results suggest that the PDAC-related decrease in ABO blood group antigens is mainly due to the decrease in H type 2 antigens. On the other hand, the present gene expression analysis of glycosyltransferases for the biosynthesis of ABO blood group antigens showed increased expression of *FUT2* and *ABO* and decreased expression of *FUT1* in the PDAC tissues. Since FUT1 and FUT2 are involved in the biosynthesis of H type 2 and H type 1 antigens, respectively (47), the present gene expression profiles suggest that the decrease in ABO blood group antigens is mainly due to the decrease in a substrate for FUT1 (i.e., H type 2 precursor). Collectively, we concluded that the lectin microarray and gene expression analyses have provided consistent results. Incidentally, many epidemiological studies have suggested associations between ABO blood group and cancer (47), and the close association has been found in the malignancy of exocrine pancreatic cancer including PDAC (48). Considering this pancreas-specific association, the present results may highlight the significance of ABO blood groups on glycoproteins in the tumor pathophysiology. A previous cohort study has reported that the secretor phenotype, which was determined by single nucleotide polymorphism of FUT2, was not an effect modifier for the association between ABO blood group alleles and pancreatic cancer risk (49). These observations are also consistent with the fact that the expression of FUT1 is relatively higher in the pancreas, based on the Tissue Atlas provided by the Human Protein Atlas project (https://www.proteinatlas.org/).

ABO blood group antigens are present in many tissues, including the pancreas (50), especially on glycolipids and *O*-glycoproteins (51, 52). We found that the *N*-glycans of basigin were affected by the PDAC-relevant aberrations in the glycosylation machinery. Notably, endogenous basigin also showed PDAC-relevant aberrant localization; basigin was localized to the basolateral membrane of pancreatic ductal epithelial cells in the non-tumor tissues, whereas it was detected throughout the cell membrane of tumor cells (Figure 5B). These results indicate the association between aberrations in glycan modification and localization of basigin. Previous studies have demonstrated the involvement of *N*-glycans in the biological functions of basigin (53, 54), thereby highlighting the importance of glycan analysis of endogenous glycoproteins enriched in clinical specimens.

The present glycan analysis strategy has several limitations. First, lectin-assisted glycan profiling provides no detailed information on the glycan structures, which must be analyzed by other analytical methods such as mass spectrometry-based approaches. Second, the present lectin microarray panel contains no lectin recognizing H type 1 derivatives and hence the present study could not evaluate the changes in these antigens, potentially affected by the increased expression levels of *FUT2* and *ABO*. This evaluation is considered important in PDAC pathology because well-known cancer-relevant glycan CA19-9 (sialyl Lewis A) is synthesized from H type 1 precursor (47) and a PDAC cell line has been demonstrated to express cell type-specific glycans recognized by recombinant BC2LCN lectin (55), which binds to Fucα1-2Galβ1-3GlcNAc (GalNAc)-containing glycans (56). Third, the present gene expression analysis focused only on limited glycosyltransferases, emphasizing the importance of the development of glyco-bioinformatic tools to provide a broad view of disease-relevant alterations in the glycosylation machinery. Finally, the present data on glycomic profiling and gene expression are obtained from tissue samples, which contain various types of cells, including normal cells to some extent. To improve this, we recently developed a method for cell type-specific tissue glycomic profiling, in which FFPE tissue sections were stained with a cell type-specific probe and the cells of interest were collected (14). This improved glycan profiling method may be combined with public databases for cell-based datasets, such as the Cancer Cell Line Encyclopedia (57). Although lectin histochemistry is useful to some extent for obtaining spatial information of target glycans, this analysis is not so quantitative and thus the staining method hardly compares the degree of staining of the lectin recognizing the glycans abundantly expressed in both tumor and non-tumor tissues (e.g., sialic acid-binding lectins). Most importantly, lectin histochemistry is no information on the glycoproteins carrying the target glycans, which is essential for the main goal of the present study (i.e., the development of novel glyco-biomarkers and therapeutic glyco-targets). To obtain information on the spatial distribution of a target glycoprotein with disease-relevant glycosylation alterations, it is required to prepare a “special probe” that recognizes multiple epitopes within both glycan structures and surrounded peptide sequences.

In conclusion, the present strategy of combining lectin microarray and public transcriptomic datasets is considered useful for evaluating the disease-relevant glycosylation alterations of endogenous glycoproteins in clinical specimens. The usefulness has been shown especially in the comparison of tissue distribution and resulting glycan modifications of the target glycoprotein. This advantage can be utilized primarily in diseases such as cancer that are accompanied by the aberrant localization of glycoproteins caused by severe impairment of tissue structures and cell polarity. Accordingly, we believe that the present strategy will facilitate the discovery of these glycoproteins for glyco-biomarker and therapeutic glyco-targets of these diseases. This strategy will be strengthened in accordance with the further development of glycan profiling methods and glyco-bioinformatic tools.

## Data Availability Statement

All datasets generated for this study are included in the article/Supplementary Material.

## Ethics Statement

The studies involving human participants were reviewed and approved by Keio University (Tokyo, Japan). The patients/participants provided their written informed consent to participate in this study.

## Author Contributions

TW, CN-O, and AM mainly performed experiments and data analysis. YM performed pathological diagnosis of clinical specimens. MI and KY provided intellectual support and expertise in the field of cancer pathology. TW, CN-O, AM, and AK wrote the manuscript. MS and AK designated all experiments in this study. All the authors contributed to the critical review of the manuscript.

### Conflict of Interest

The authors declare that the research was conducted in the absence of any commercial or financial relationships that could be construed as a potential conflict of interest.

## Footnotes

^1^https://www.genome.jp/kegg/pathway.html

^2^https://xenabrowser.net/datapages/
