# Supplementary material for: Discovery of Pancreatic Ductal Adenocarcinoma-Related Aberrant Glycosylations: A Multilateral Approach of Lectin Microarray-Based Tissue Glycomic Profiling With Public Transcriptomic Datasets
Source: Front Oncol. 2020 Mar 13;10:338. doi: 10.3389/fonc.2020.00338 (PMC7082313; doi:10.3389/fonc.2020.00338)
Supplement: Supplementary file 1 [file Image_1.pdf]

Supplementary Table 1. Abbreviations and carbohydrate specificities of 45 lectins on the LecChip Ver.1.0

Lectin	Origin	Reactivity[a]
LTL	*Lotus tetragonolobus*	Fucα1,3-GlcNAc; Sialyl Lewis x; Lewis x
PSA	*Pisum sativum*	Fucα1,6-GlcNAc; α-Man
LCA	*Lens culinaris*	Fucα1,6-GlcNAc; α-Man; α-Glc
UEA-I	*Ulex europaeus*	Fucα1,2-Galβ1,4-GlcNAc (Type II, H antigen)
AOL	*Aspergillus oryzae*	Terminal α-Fuc; Sialyl Lewis x; Lewis x
AAL	*Aleuria auranti*	Terminal α-Fuc; Sialyl Lewis x; Lewis x
MAL-I	*Maackia amurensis*	Siaα2,3-Gal
SNA	*Sambucus nigra*	Siaα2,6-Gal/GalNAc
SSA	*Sambucus sieboldiana*	Siaα2,6-Gal/GalNAc
TJA-I	*Trichosanthes japonica*	Siaa2,6-Galβ1,4-GlcNAc
PHA-L	*Phaseolus vulgaris*	Tri/tetra-antennary complex-type *N*-glycan
ECA	*Erythrina cristagalli*	Lac/LacNAc
RCA120	*Ricinus communis*	Lac/LacNAc
PHA-E	*Phaseolus vulgaris*	Bi-antennary complex-type *N*-glycan with outer Gal; Bisecting GlcNAc
DSA	*Datura stramonium*	Chitin; PolyLacNAc; LacNAc
GSL-II	*Griffonia simplicifolia*	Agalactosylated tri/tetra antennary *N*-glycan
NPA	*Narcissus pseudonarcissus*	High-Mannose including Manα1,6-Man
ConA	*Canavalia ensiformis*	High-Mannose including Manα1,6 (Manα1,3)-Man
GNA	*Galanthus nivalis*	High-Mannose including Manα1,3-Man
HHL	*Hippeastrum hybrid*	High-Mannose including Manα1,3-Man or Manα1,6-Man
ACG	*Agrocybe cylindracea*	Siaα2,3-Galβ1,4-GlcNAc
TxLC-I	*Tulipa gesneriana*	Manα1,3(Manα1,6)-Man; Bi/tri-antennary complex-type *N*-glycan; GalNAc
BPL	*Bauhinia purpurea*	Galβ1,3-GalNAc; Tri/tetra-antennary complex-type N-glycan with outer Gal
TJA-II	*Trichosanthes japonica*	Fucα1,2-Galβ1/GalNAcβ1; Tri/tetra-antennary complex-type *N*-glycan with outer Gal
EEL	*Euonymus europaeus*	Galα1,3[Fucα1,2-Gal] (B antigen); Galα1,3-Gal
ABA	*Agaricus bisporus*	Galβ1,3-GalNAcα-Thr/Ser (T antigen); Sialyl-T
LEL	*Lycopersicon esculentum*	Chitin; PolyLacNAc
STL	*Solanum tuberosum*	Chitin; PolyLacNAc
UDA	*Urtica dioica*	Chitin; PolyLacNAc
PWM	*Phytolacca Americana*	Chitin; PolyLacNAc
Jacalin	*Artocarpus integrifolia*	Galβ1,3-GalNAcα-Thr/Ser (T); GalNAcα-Thr/Ser (Tn antigen)
PNA	*Arachis hypogaea*	Galβ1,3-GalNAcα-Thr/Ser (T)
WFA	*Wisteria floribunda*	GalNAcβ1,4-GlcNAc (LacdiNAc); Terminal GalNAc
ACA	*Amaranthus caudatus*	Galβ1,3-GalNAcα-Thr/Ser (T)
MPA	*Maclura pomifera*	Galβ1,3-GalNAcα-Thr/Ser (T); GalNAcα-Thr/Ser (Tn)
HPA	*Helix pomatia*	Terminal GalNAc
VVA	*Vicia villosa*	α-, β-linked terminal GalNAc; GalNAcα-Thr/Ser (Tn)
DBA	*Dolichos biflorus*	GalNAcα-Thr/Ser (Tn); GalNAcα1,3-GalNAc
SBA	*Glycine max*	Terminal GalNAc (especially GalNAcα1,3-Gal)
Calsepa	*Calystegia sepium*	Man; Maltose
PTL-I	*Psophocarpus tetragonolobus*	α-GalNAc; Gal
MAH	*Maackia amurensis*	Siaα2,3-Galβ1,3(Siaα2,6)-GalNAc (disialyl-T)
WGA	*Triticum aestivum*	Chitin; Multivalent Sia
GSL-I-A4	*Griffonia simplicifolia*	α-GalNAc; GalNAcα-Thr/Ser (Tn)
GSL-I-B4	*Griffonia simplicifolia*	α-Gal

[a]These reactivity of lectins were based on Lectin Frontier Database (LfDB; https://acgg.asia/lfdb2/).

Supplementary Table 2. Clinicopathologic features for 14 PDAC cases.

Age	[Range]
Mean, years	67.4 [55-84]
Gender	
Male	10
Female	4
Blood type	
O	3
A	8
B	2
AB	1
Tumor location	
Head	4
Body	4
Tail	6
Tumor size	
Mean, cm	3.0 [1.6-5.7]
Tumor grade	
G1	1
G2	10
G3	3
Stage (AJCC 8th edition)	
I	3
II	10
III	1

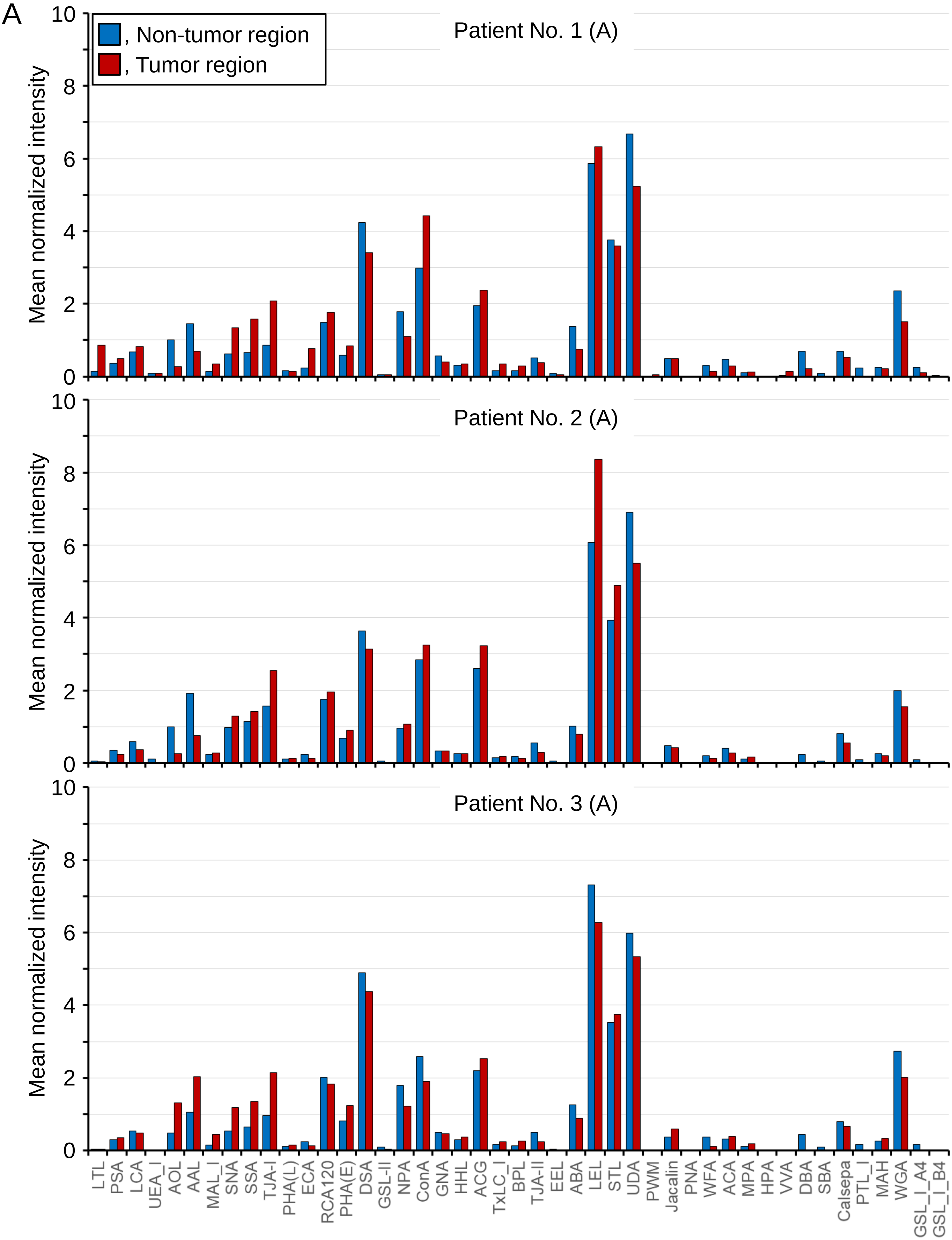

Supplementary Figure 1. The tissue glycomic profiles of 14 PDAC cases.
(A) Blood type A (Patient No. 1–8), **(B)** Blood type B (No. 9–10), **(C)** Blood type O (No. 11–13), and **(D)** Blood type AB (No.14). Blue, non-tumor regions; Red, tumor regions.

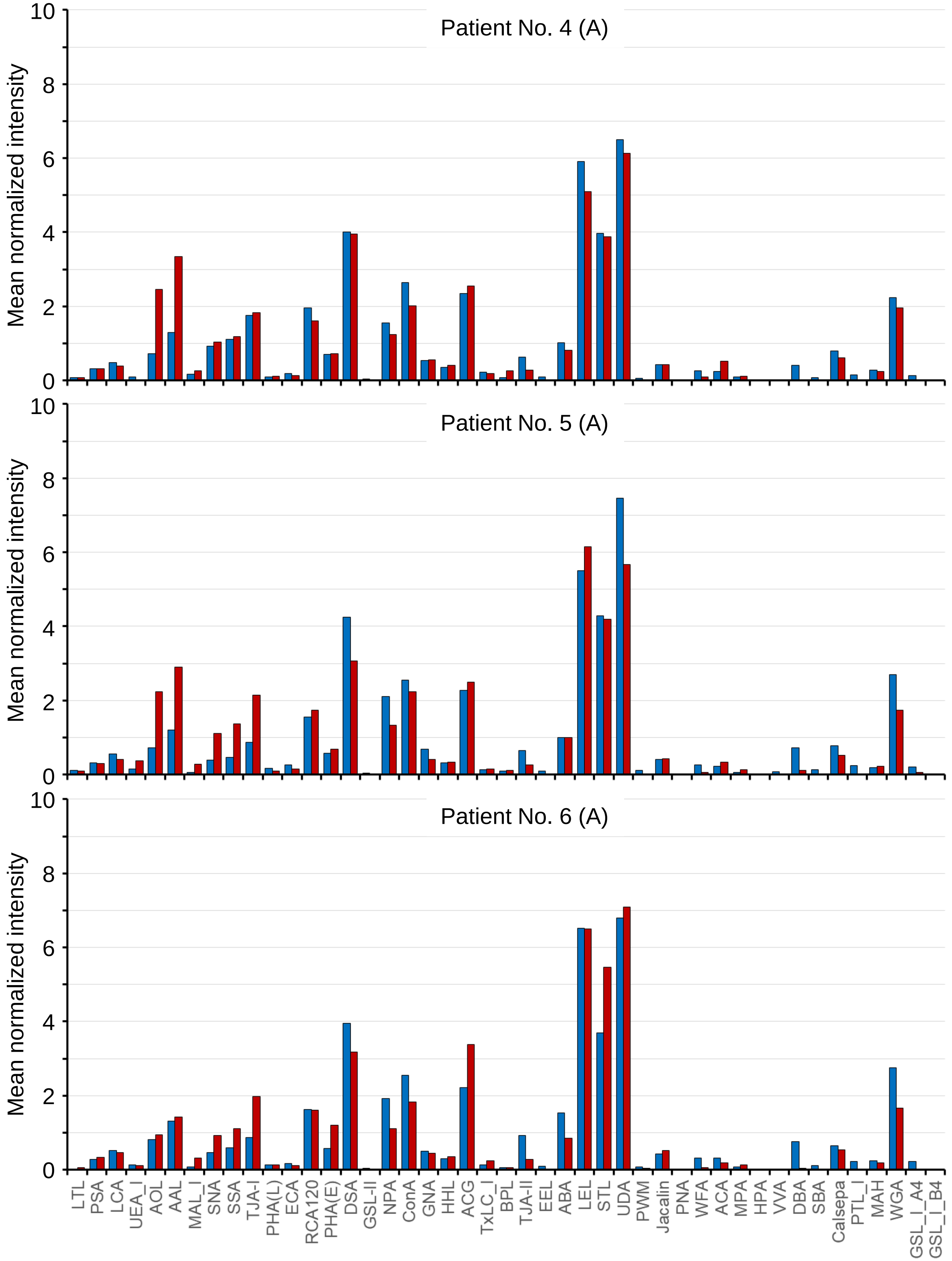

Supplementary Figure 1. Continued

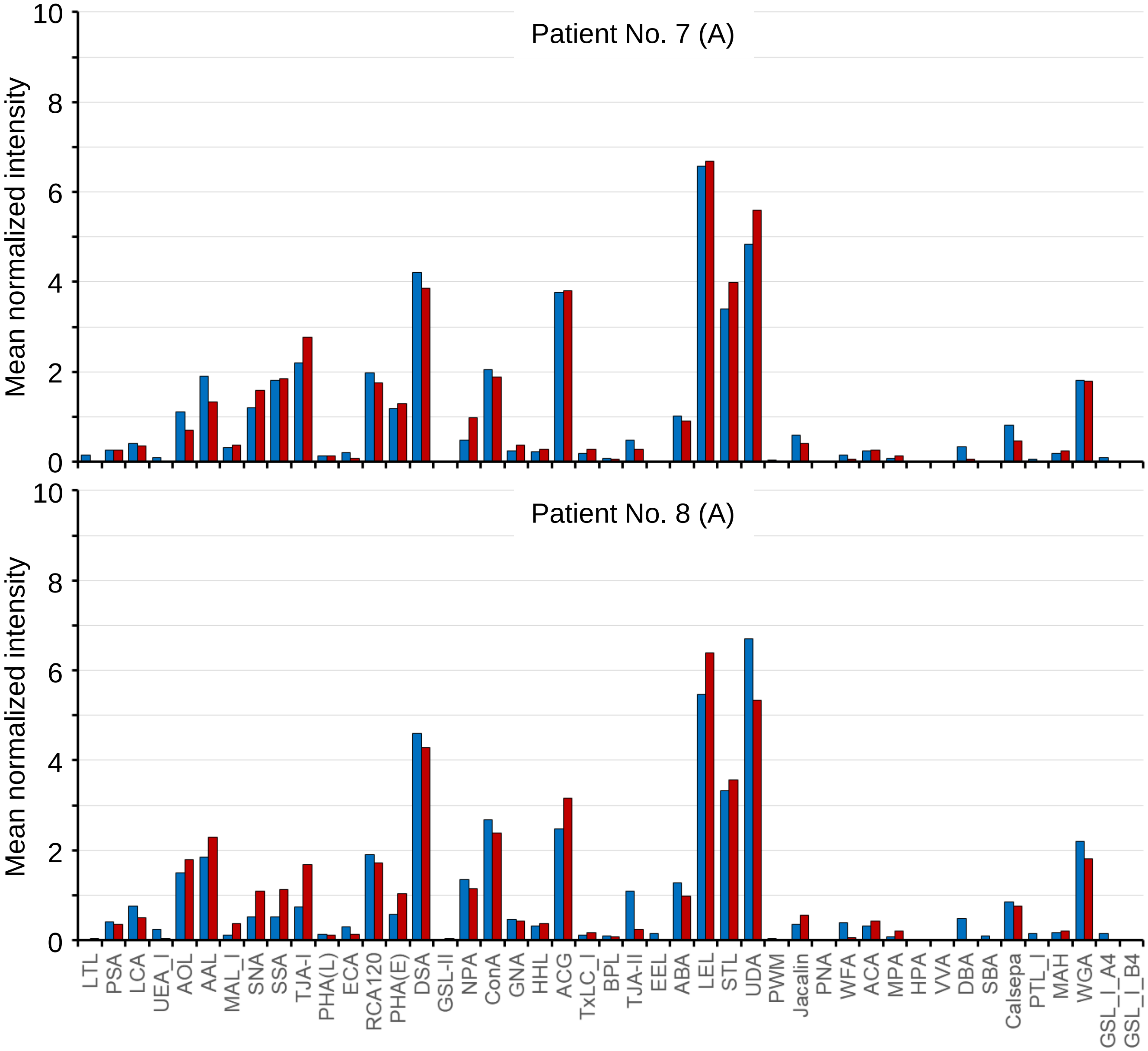

Supplementary Figure 1. Continued

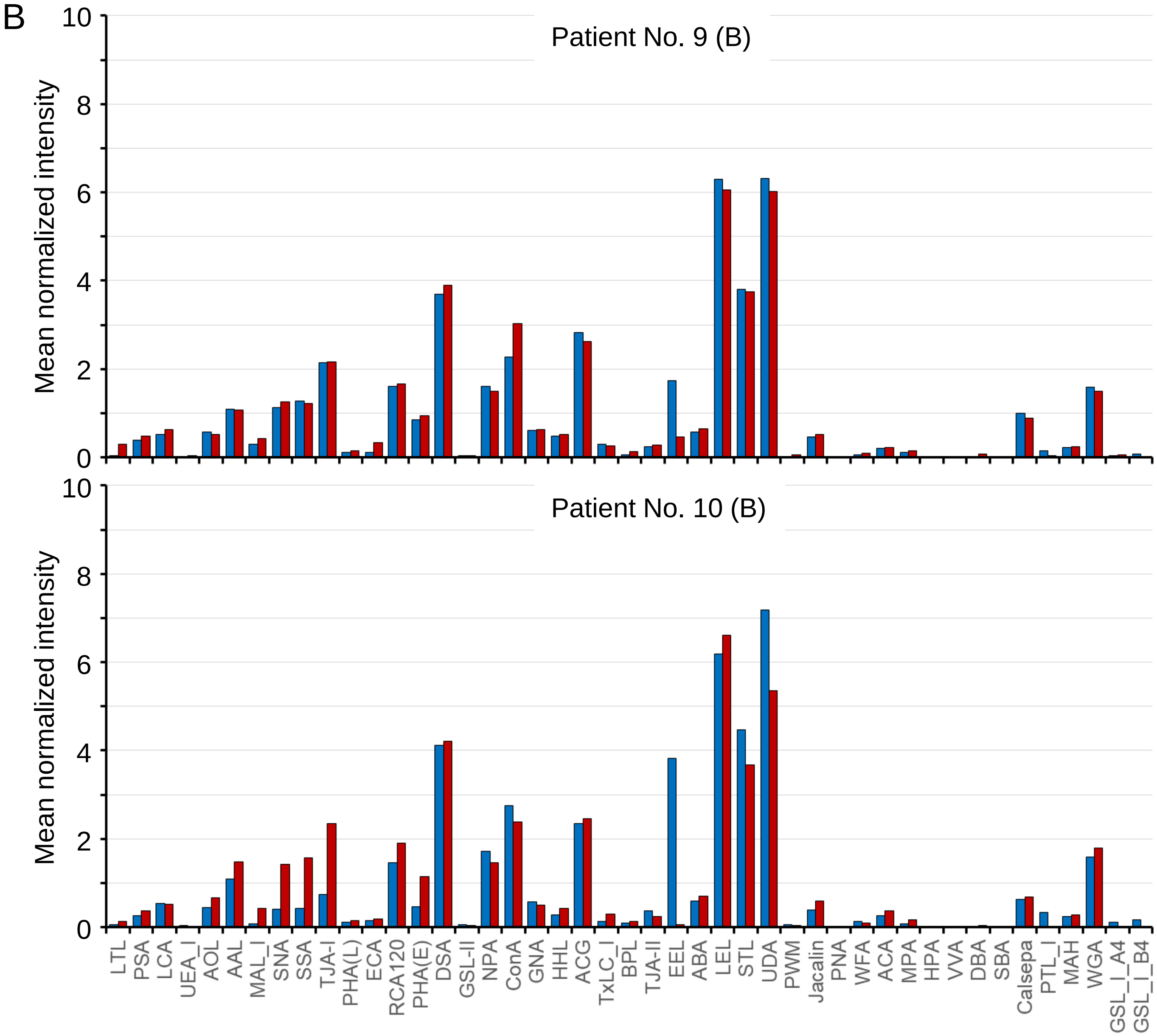

Supplementary Figure 1. Continued

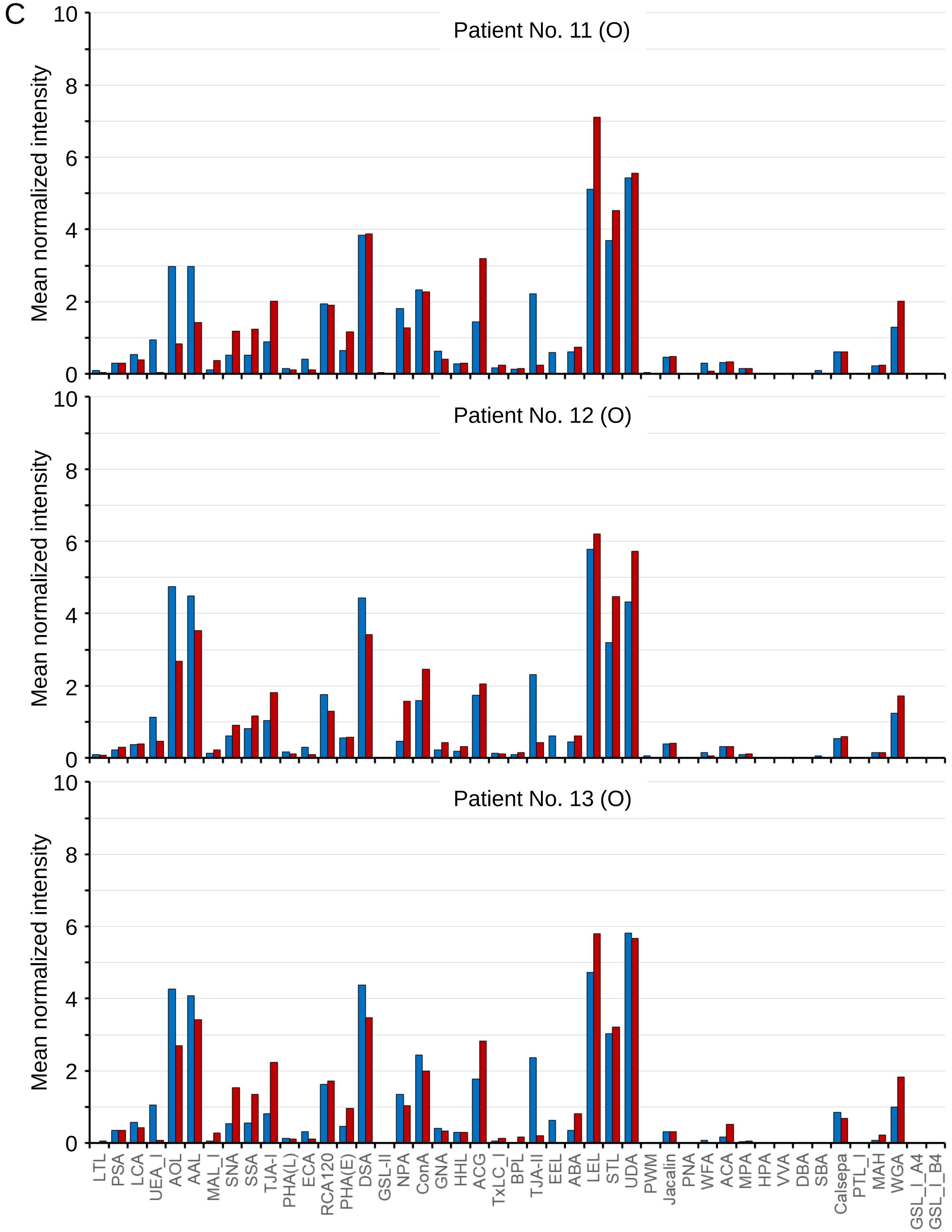

Supplementary Figure 1. Continued

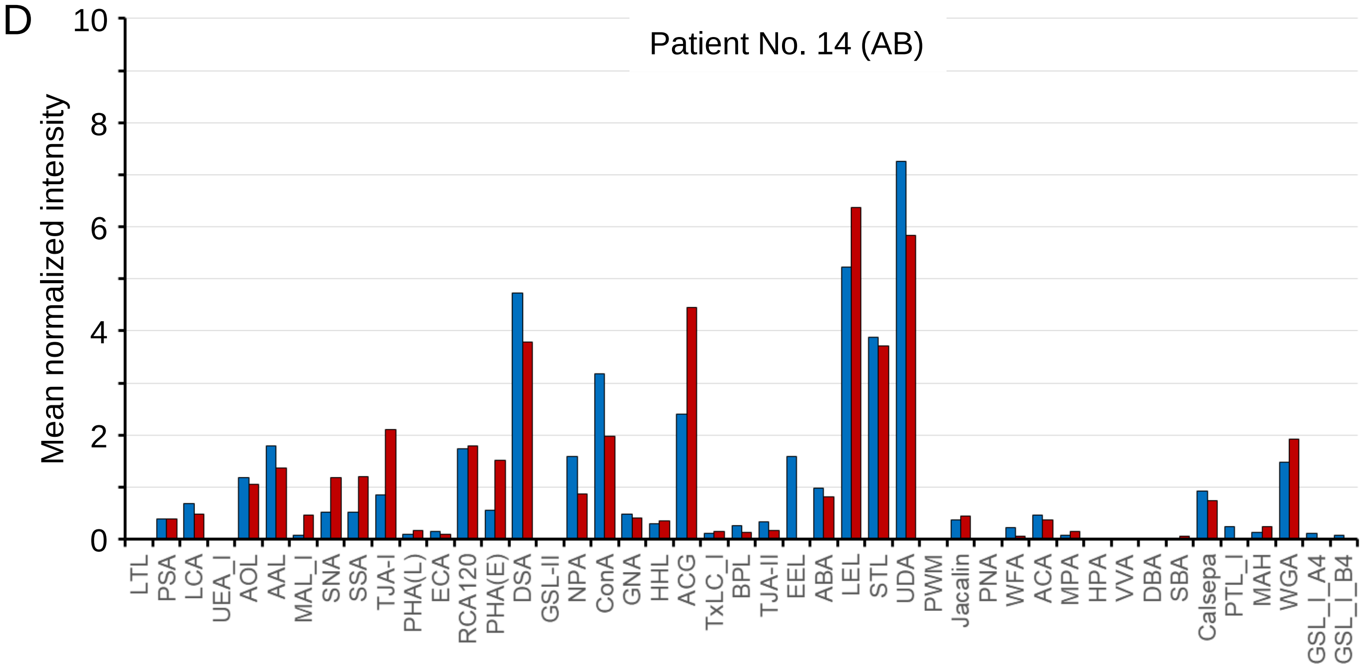

Supplementary Figure 1. Continued

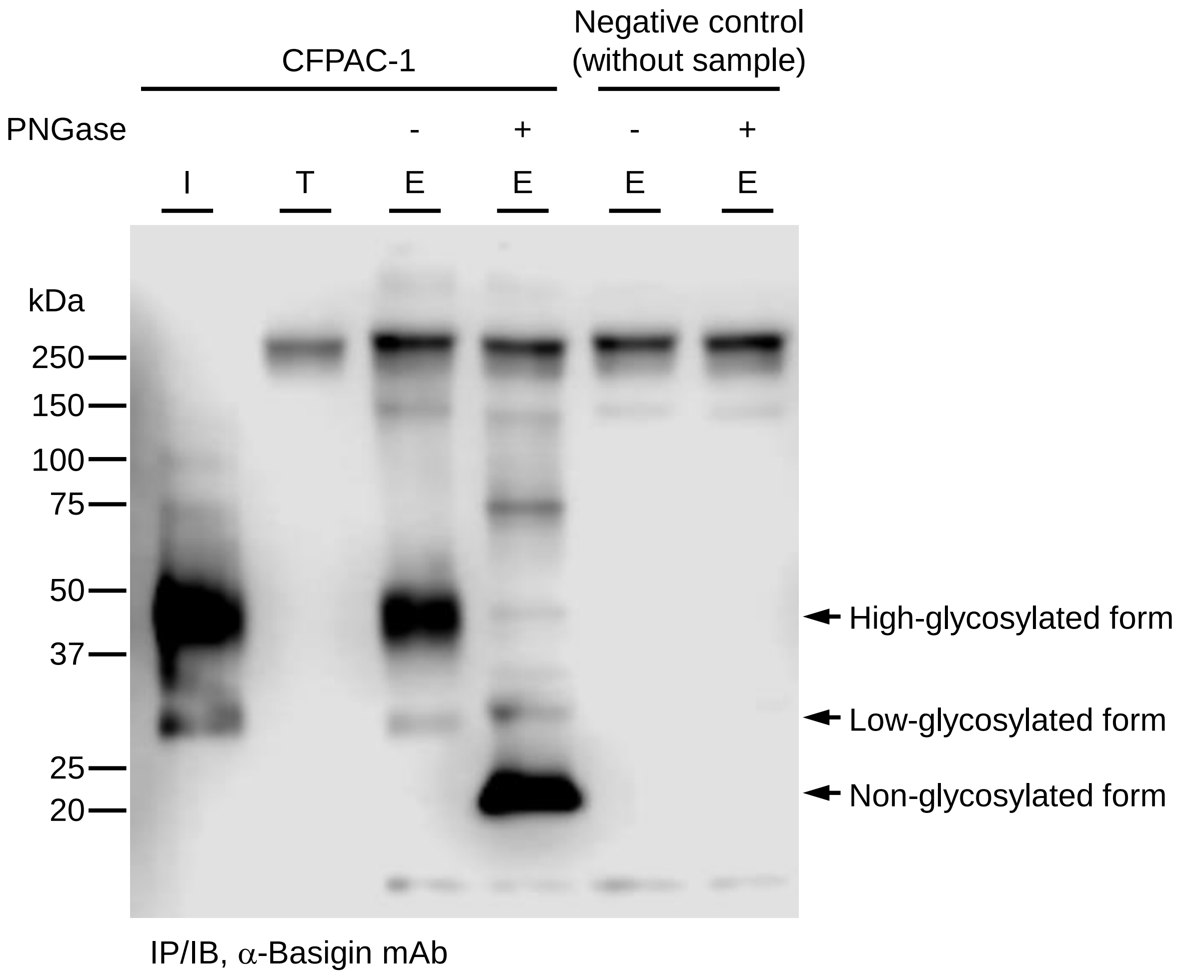

Supplementary Figure 2. Western blot analysis for basigin immunoprecipitated from the cell membrane fractions of CFPAC-1.

This *N*-glycoprotein was detected as high- and low-glycosylated forms in the input (I) and elution fraction (E) and not detected in the through fraction (T). After treatment of the immunoprecipitated basigin with peptide:*N*-glycanase (PNGase), the high-glycosylated form was decreased, and the non-glycosylated form became dominant.

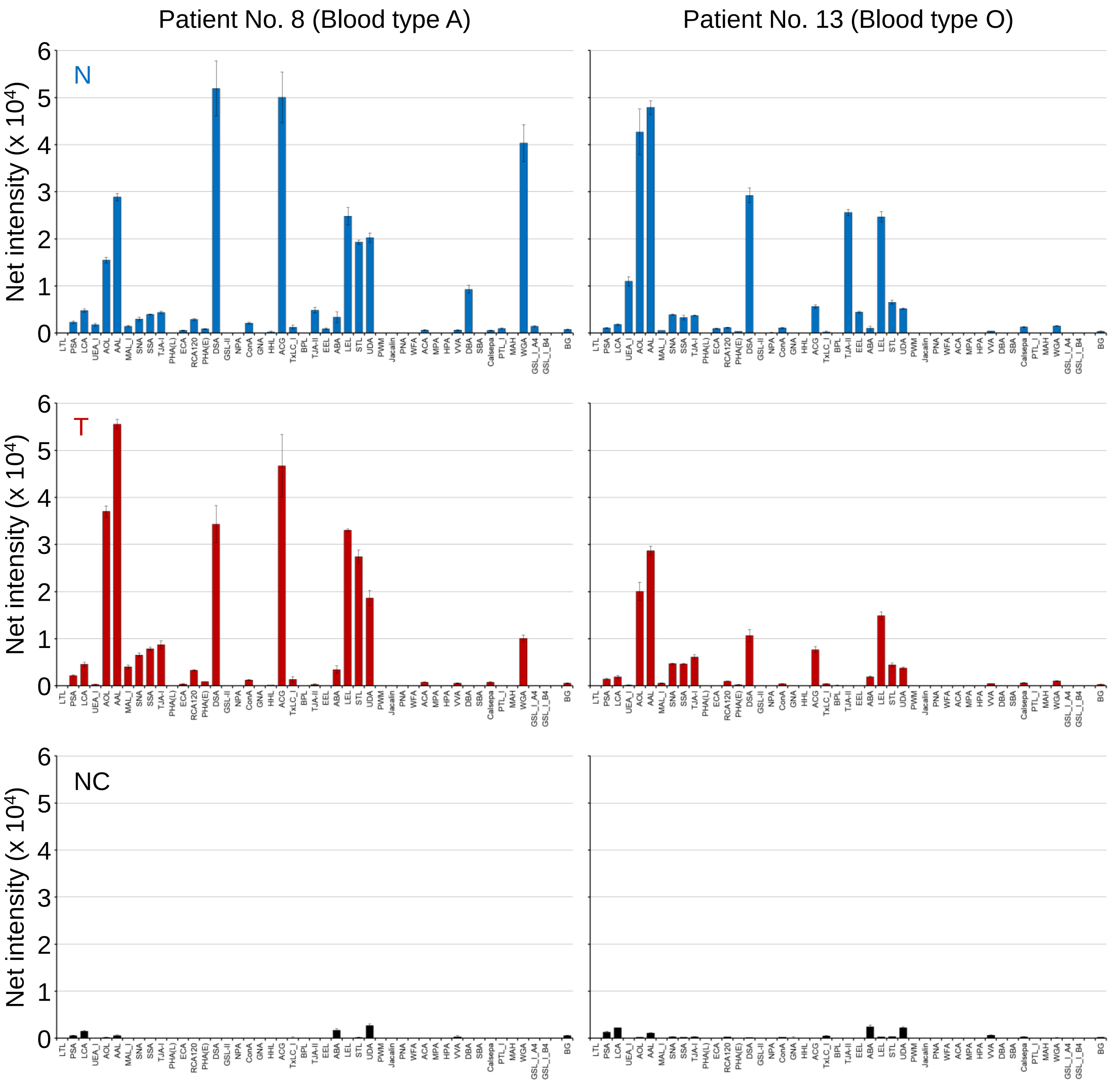

Supplementary Figure 3. The glycan profiling of immunoprecipitated basigin from tissue extracts. The typical glycan profiles of immunoprecipitated basigin indicated in Figure 5C. The signal values were applicable for differential analysis between tumor (T) and non-tumor (N) regions. There were no significant noises derived from the overlay antibody against basigin in the negative control (NC) without tissue samples.

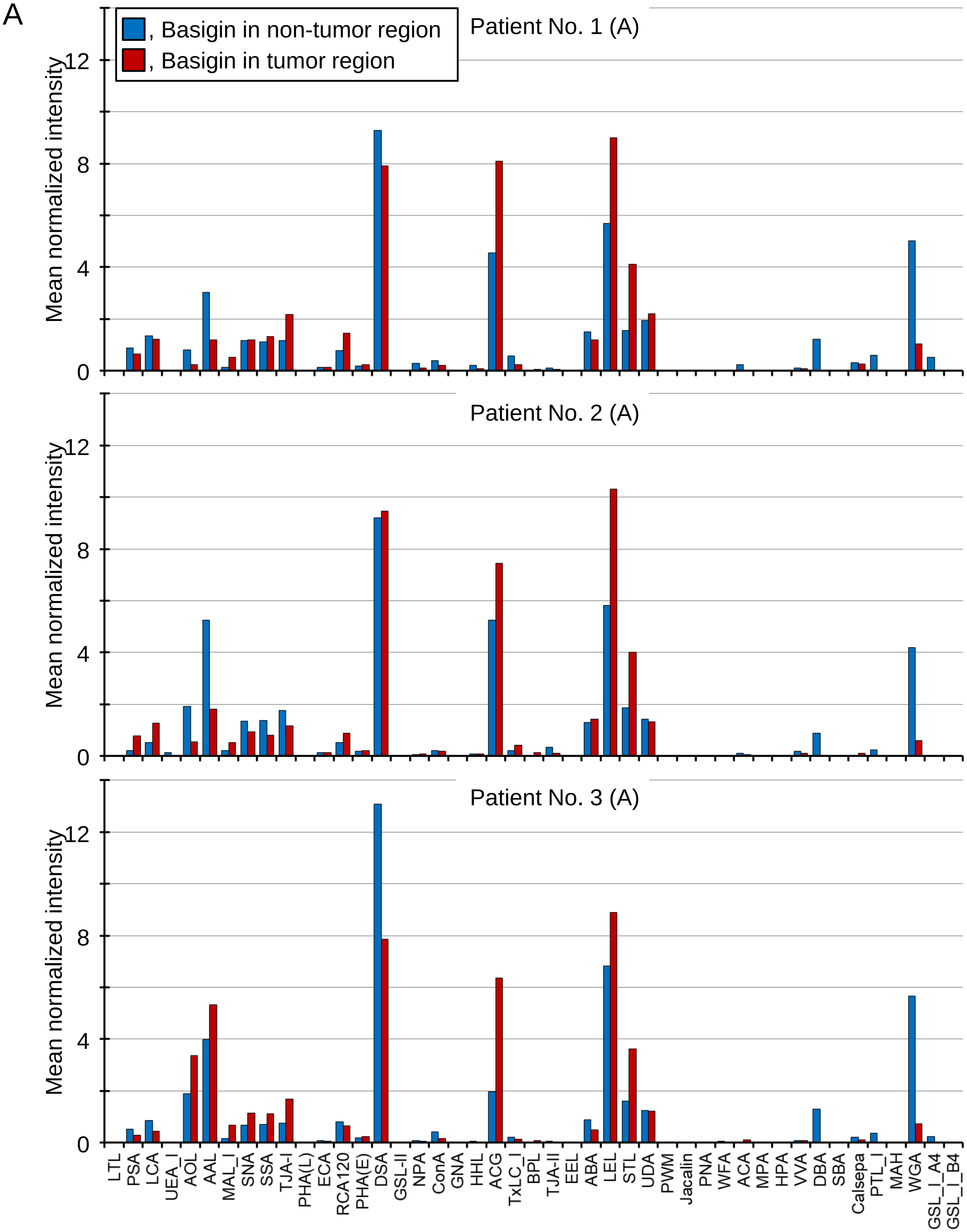

Supplementary Figure 4. The glycan profiles of basigin immunoprecipitated from 14 PDAC tissues. **(A)** Blood type A (Patient No. 1−8), **(B)** Blood type B (No. 9−10), **(C)** Blood type O (No. 11−13), and **(D)** Blood type AB (No.14). Blue, basigin obtained from non-tumor regions; Red, basigin obtained from tumor regions.

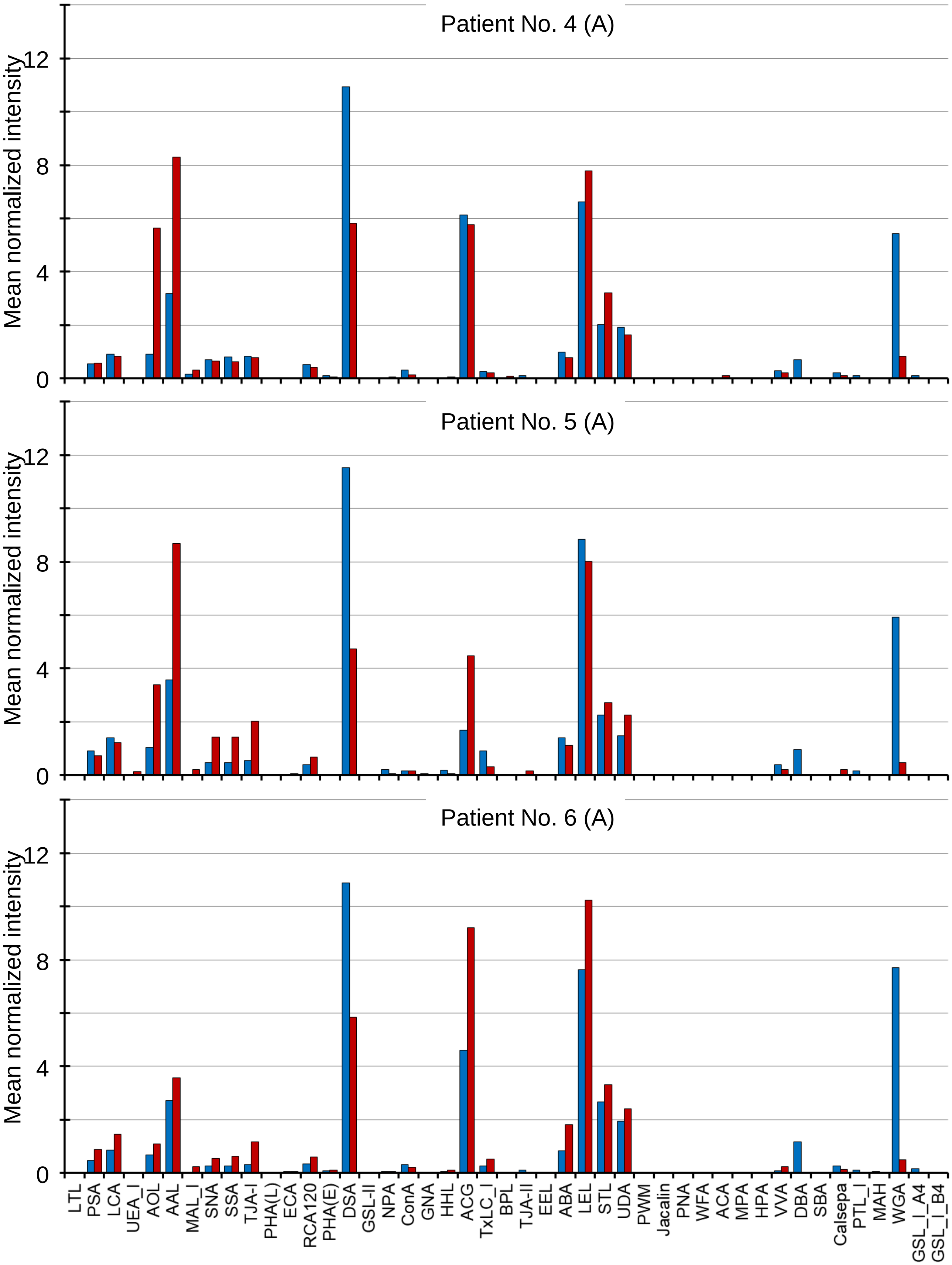

Supplementary Figure 4. Continued

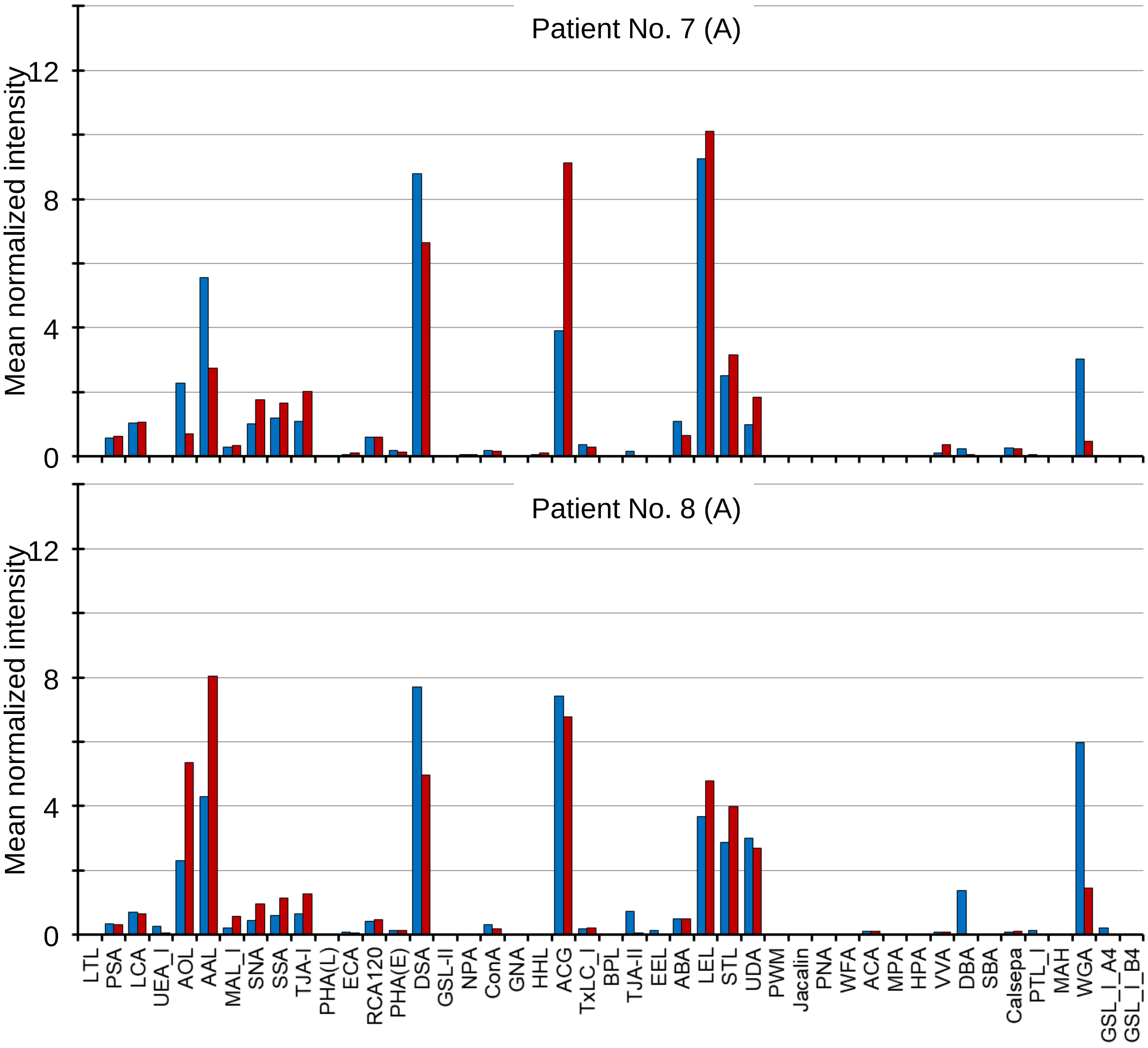

Supplementary Figure 4. Continued

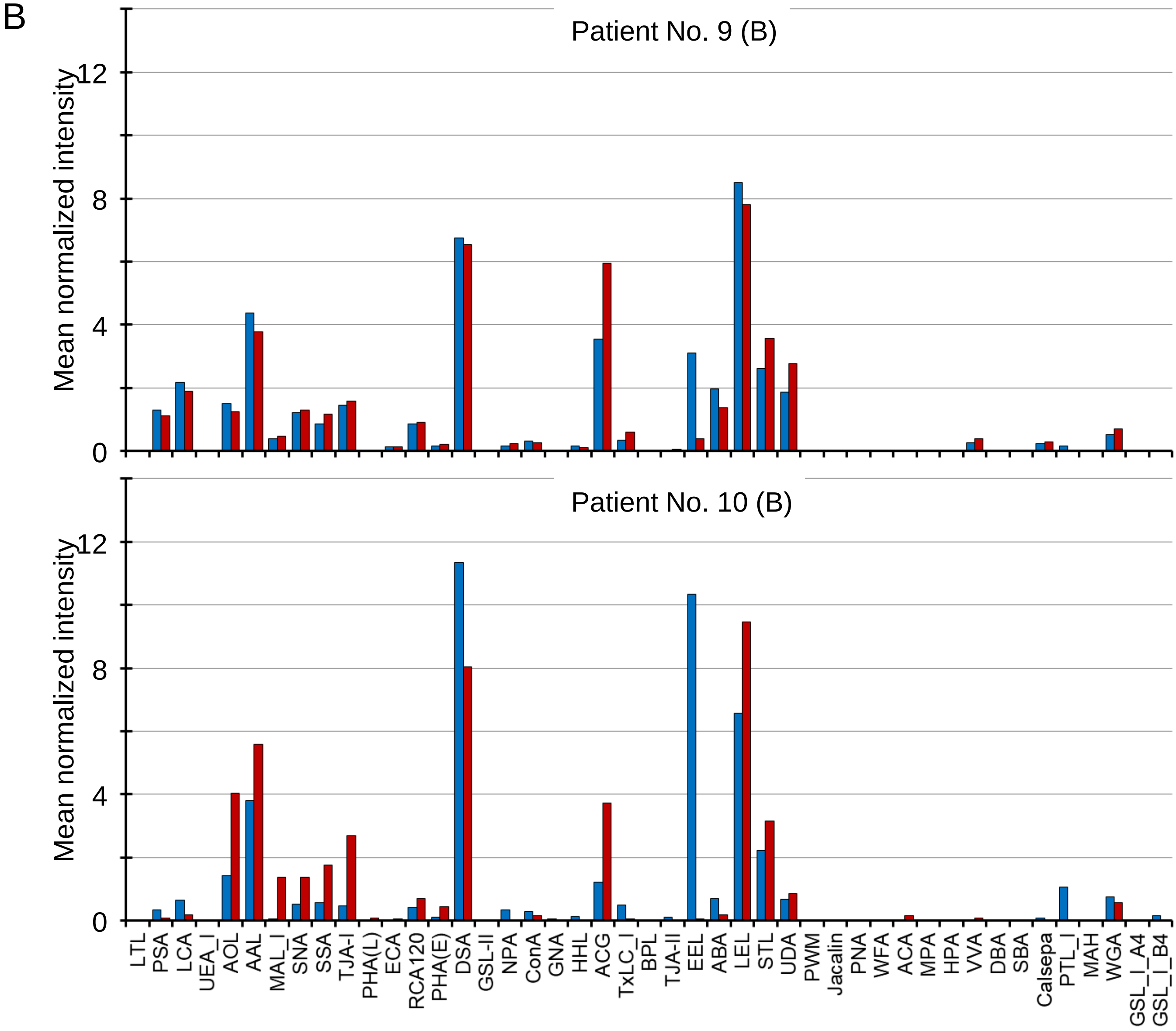

Supplementary Figure 4. Continued

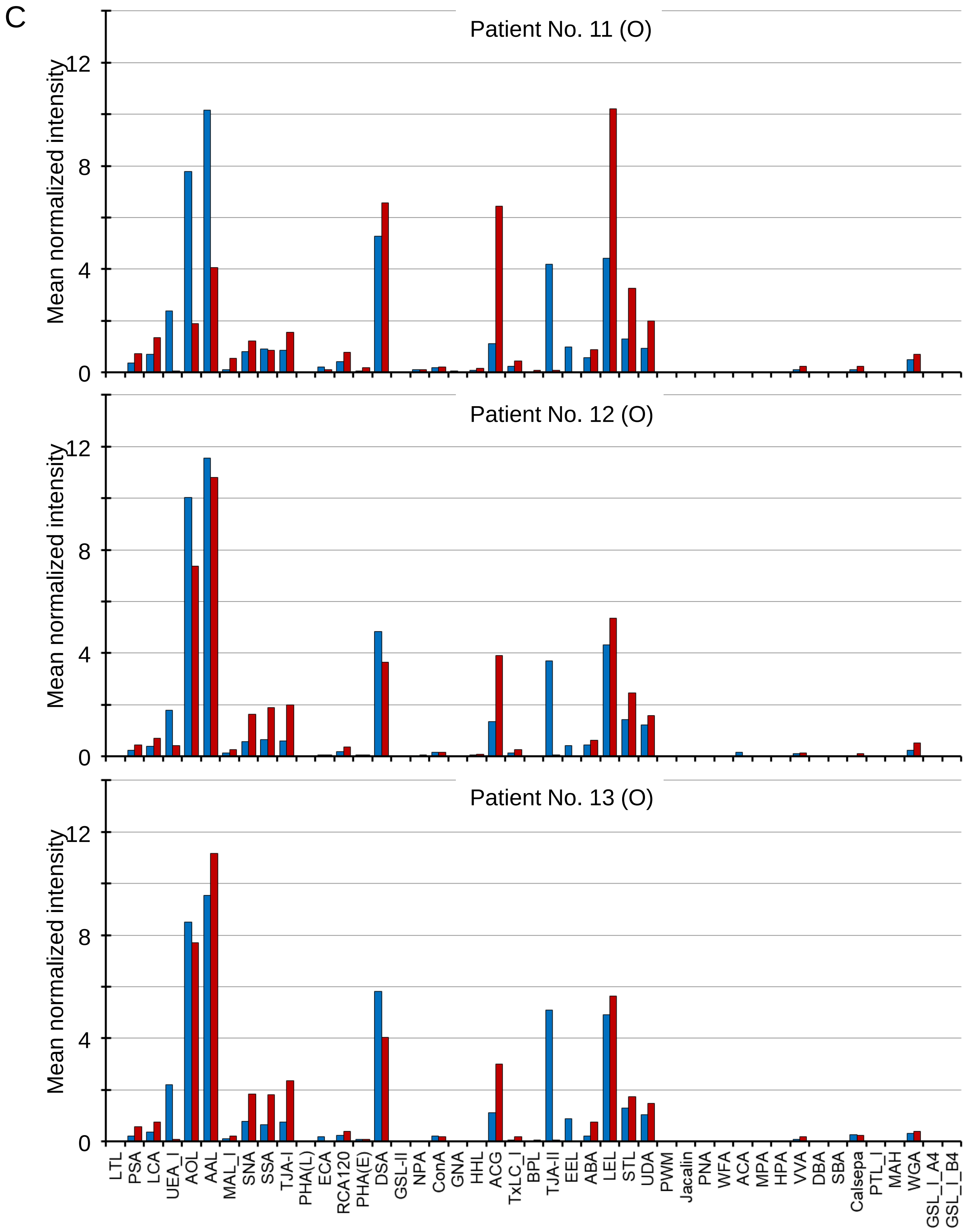

Supplementary Figure 4. Continued

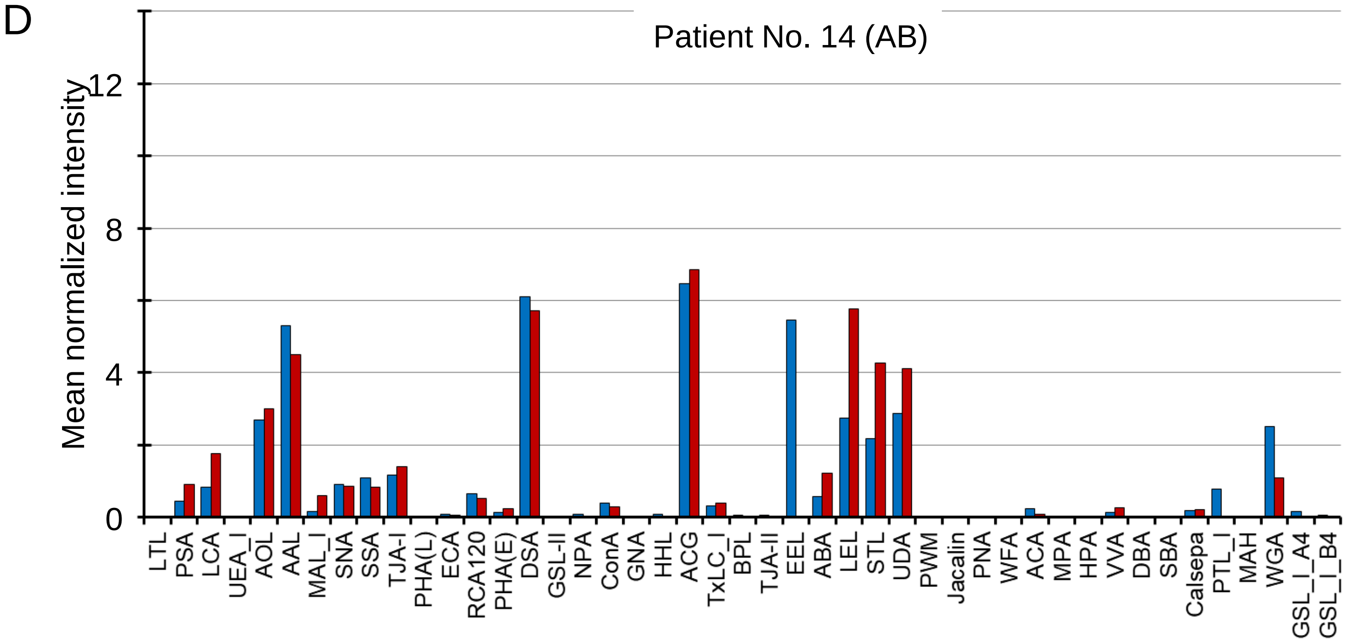

Supplementary Figure 4. Continued
